# GASOLINE: detecting germline and somatic structural variants from long-reads data

**DOI:** 10.1038/s41598-023-48285-0

**Published:** 2023-11-27

**Authors:** Alberto Magi, Gianluca Mattei, Alessandra Mingrino, Chiara Caprioli, Chiara Ronchini, Gianmaria Frigè, Roberto Semeraro, Marta Baragli, Davide Bolognini, Emanuela Colombo, Luca Mazzarella, Pier Giuseppe Pelicci

**Affiliations:** 1 Department of Information Engineering, University of Florence, 50100 Florence, Italy; 2 Institute for Biomedical Technologies, National Research Council, Segrate, Milan, Italy; 3 Department of Experimental and Clinical Medicine, University of Florence, Florence, Italy; 4 Department of Experimental Oncology, IEO European Institute of Oncology IRCCS, Milan, Italy; 5 Department of Oncology and Hemato-Oncology, University of Milan, Milan, Italy

**Keywords:** Computational biology and bioinformatics, Genome informatics

## Abstract

Long-read sequencing allows analyses of single nucleic-acid molecules and produces sequences in the order of tens to hundreds kilobases. Its application to whole-genome analyses allows identification of complex genomic structural-variants (SVs) with unprecedented resolution. SV identification, however, requires complex computational methods, based on either read-depth or intra- and inter-alignment signatures approaches, which are limited by size or type of SVs. Moreover, most currently available tools only detect germline variants, thus requiring separate computation of sample pairs for comparative analyses. To overcome these limits, we developed a novel tool (Germline And SOmatic structuraL varIants detectioN and gEnotyping; GASOLINE) that groups SV signatures using a sophisticated clustering procedure based on a modified reciprocal overlap criterion, and is designed to identify germline SVs, from single samples, and somatic SVs from paired test and control samples. GASOLINE is a collection of Perl, R and Fortran codes, it analyzes aligned data in BAM format and produces VCF files with statistically significant somatic SVs. Germline or somatic analysis of 30$\times$ sequencing coverage experiments requires 4–5 h with 20 threads. GASOLINE outperformed currently available methods in the detection of both germline and somatic SVs in synthetic and real long-reads datasets. Notably, when applied on a pair of metastatic melanoma and matched-normal sample, GASOLINE identified five genuine somatic SVs that were missed using five different sequencing technologies and state-of-the art SV calling approaches. Thus, GASOLINE identifies germline and somatic SVs with unprecedented accuracy and resolution, outperforming currently available state-of-the-art WGS long-reads computational methods.

## Introduction

Structural variants (SVs) are genomic alterations typically defined (and somewhat arbitrarily) as DNA segments larger than 50 bp that can be deleted, duplicated, inserted, inverted or translocated compared to a reference genome. SVs are among the main sources of germline genomic variation in humans and can be associated with several diseases, including type I diabetes^1^, cardiovascular disease^2^, neurological disorders^3^ and cancer^4^. Moreover, somatic SVs acquired by cancer genomes are known drivers of carcinogenesis and their detection is essential for either diagnosis or treatment stratification in at least 30% of cancer patients^5^.

In the past decade second-generation sequencing (SGS) technologies, based on high-throughput short-read generation^6^, together with the development of powerful computational tools, have revolutionized our capability to study structural variations (SVs) of any size, from small insertions/deletions to large CNVs, with unprecedented accuracy in determining position and orientation^7^. However, it is apparent that the short reads (100–400 bp) generated by these platforms are insufficient to confidently detect variants larger than 50 bp, in particular those in the range [50, 1000] bp^8^. Moreover, Kosugi et al.^9^ performing a comprehensive comparison of 69 available algorithms for SV detection from short reads, found that all these tools obtain low values of recall (between 20 and 40%) demonstrating the limits of this technology in detecting SVs.

The paste decade has seen the emergence of a third generation of sequencing technologies based on single-molecule real-time (Pacific Biosciences, PacBio)^10^ and nanopore sequencing (Oxford Nanopore Technologies, ONT)^11^, which interrogate single molecule of DNA and are capable to produce sequences much longer than those generated by SGS methods.

Chaisson et al.^8^ and Huddleston et al.^12^, by using deep PacBio sequencing data from two haploid human genomes, resolved the complete sequence of a large amount of SVs, showing that around 80% of these variants were missed by SGS data (with the greatest increase in sensitivity occurring for events smaller than 5 kb, in size). More recently, Zhao et al.^13^ showed that only a limited subset of SVs overlapped between short-read and long-read technologies (66.8% of short- and 33.5% of long-reads events). Moreover, the SV class strongly impacted concordance: 60.6% of short- and 48.7% of long-reads deletions demonstrated overlap as compared with 81.7% of short- and 24.1% of long-reads insertions. These results demonstrated that the use of long read data can definitively enlarge the spectrum of detectable genetic variants, becoming the cutting-edge approach for the study of complex genomic structures.

Identification of SVs from long-reads data requires complex computational methods, which are based on either read-depth (depth of coverage, DOC) or intra- and inter-alignment SV signatures (split-read alignments) approaches^14^. While the DOC approach is limited to the identification of large deletions and duplications ($>100$ kb)^15^, gapped alignment methods allow detection of deletions, inversion and translocations of any size, with insertions and duplications limited by read length^14^.

All split-read approaches consist of complex procedures in which the genomic coordinates of SV signatures are clustered on the basis of their reciprocal overlap to find groups of similar signatures that support the same SV. This step is critical for recovering all the signatures generated by each SV. Incomplete recovery can in fact lead to underestimation of the allelic fraction and genotyping errors. Signature clustering becomes even more critical when comparing datasets of matched test and control samples for somatic variant detection, where missing of some SV signatures in the control sample can lead to the calling false positive somatic variants.

At present, most of the currently available tools, such as SVIM^16^, CuteSV^17^ and Sniffles^18^, only detect germline variants, using SV-signature clustering procedures that are based on classical reciprocal overlap. Remarkably, the standard approach for the detection of somatic variants consists in the application of these methods separately on each of the paired samples (test and control), discarding SVs with supporting reads in the control sample^19^.

To overcome the limits of currently available methods, we developed GASOLINE (Germline And SOmatic structuraL varIants detectioN and gEnotyping) tool, a software that, exploiting a novel reciprocal overlap measure to cluster SV signatures is capable to detect germline SVs from the analysis of single samples as well as somatic SVs from the comparison of test and matched-normal samples. We tested our novel tool on synthetic and real long reads datasets and demonstrated its potential to detect germline and somatic SVs with unprecedented accuracy and resolution.

## Results

### Germline GASOLINE

When sequencing data are aligned to a reference genome, in principle, each SV subtype generates a typical pattern of mapped reads, named SV signature, which is then used to identify the underlying alteration. These signatures can be classified in two distinct categories: gapped alignment or split read alignments. Alignment algorithms use gap penalty^20^ to account for genomic differences (alterations) occurring from insertions or deletions in the sequences. For example, a deletion, that is a lack of a sequence, generates a gap in the alignment of the read relative to a reference, while an insertion creates a gap in the alignment of the reference relative to the read. When genomic differences are too large and exceed gap penalty, mapping algorithms generate split-alignment, in which consecutive segments of the query sequence are mapped to disjoint regions in the reference and can have discordant orientation.

While gapped alignment generates two signature categories (insertions and deletions), the signatures arising from split reads can recognize, in principle every type of alteration: (i) two consecutive segments mapping far apart with the same or opposite orientations define, respectively, deletion or inversion signatures; (ii) overlapping coordinates define a duplication signature; (iii) a read splitted in three segments, with the first and third segments closely mapped, define an insertion signature; (iv) finally, when two consecutive segments mapped on different chromosomes define a translocation signature (Supplementary Fig. S1).

The first critical step of SVs detection consists in finding and grouping, all the signatures generated by each genomic alteration. From a computational point of view this step consists in clustering the genomic coordinates of SV signatures to find groups of intervals with large reciprocal overlap.

Owing to the high error rate, the alignment of long read data can be very noisy and the genomic coordinates of SV signatures generated by the same event can be imprecise and have variances of tens of bp. In this situation, identifying and clustering SVs signatures generated by small events (50–500 bp) require small reciprocal overlap that takes into consideration noisy and imprecise alignments, while large SVs (tens or hundreds of kb), less affected by error rate, needs large reciprocal overlap to prevent the inclusion of signatures arising from other events.

Thus, the use of standard reciprocal overlap criteria can underestimate or completely miss signatures of small SVs (using large reciprocal overlap), or include wrong signatures in the identification of large SVs (using small reciprocal overlap may include signatures of other SVs).

To overcome the limits of classical reciprocal overlap we introduced a novel normalized reciprocal overlap (NRO) criterion that allows grouping both small and large SV signatures with high accuracy, thus reducing the effect of imprecise alignment.

NRO mitigates the effect of imprecise alignment by taking into account both overlapping and non-overlapping regions of two SV signatures with the following formula:

$$\begin{aligned} NRO_{ij}=2 \cdot \frac{Overlap}{Li+Lj}-\frac{NO_i+NO_j}{NO_{Norm}} \end{aligned}$$

where $L_i$ and $L_j$ are the the size of the two signatures, *Overlap* and *NO* are the size of the overlapping and non-overlapping regions between the two signatures respectively and $NO_{Norm}$ is a normalization factor. The first term represents the classical reciprocal overlap between two signatures, while the second term allows mitigation of the effect of imprecise alignments for small variants and prevents the inclusion of erroneous signatures in large SVs.

**Figure 1:**
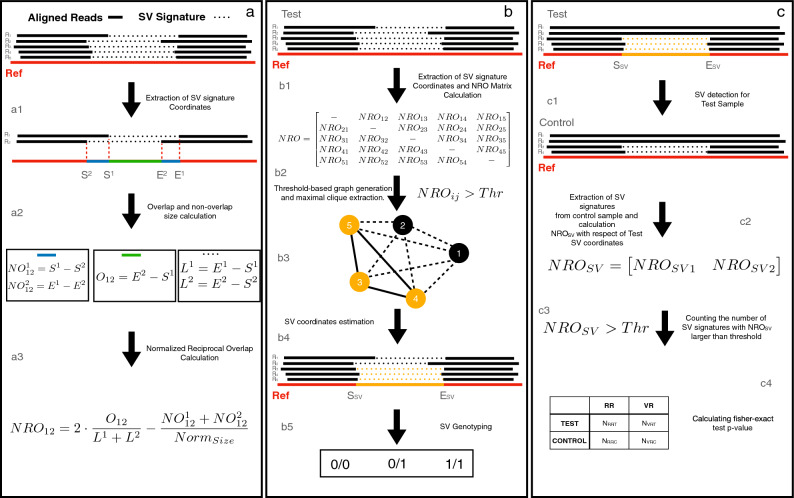
GASOLINE workflow. Panel (**a**) shows the steps to calculate NRO for a pair of SV signatures coordinates. Once the gap- and split-alignments coordinates ($S_i$ and $E_i$ for $i,j=[1,2]$) have been extracted from each read (**a1**), these are used to calculate the size of non-overlapping ($NO^{1}_{12}$ and $NO^{2}_{12}$, blue lines) and overlapping ($O_{12}$) segments for each pair of signatures (**a2**). $NO^{1}_{12}$, $NO^{2}_{12}$, $O_{12}$ and the total size of the two intervals ($L^1$ and $L^2$) are then used to calculate the $NRO_{12}$ coefficient (**a3**). In panel (**b**) is reported the workflow followed by GASOLINE for the detection of germline SVs in a sample. After signatures extraction, the tool calculates the $NRO_{ij}$ between all the signature pairs and generates an NRO matrix (**b1**) that is used as adjacency matrix to create an undirected graph by filtering out $NRO_{ij}$ values smaller than a predefined threshold (continuous edges represent $NRO_{ij}>NRO_{thr}$, while dotted edges $NRO_{ij}<NRO_{thr}$ (**b2**). The undirected graph is then analyzed with the Eppstein–Löffler–Strash algorithm to extract maximal cliques that represent clusters of SV signatures that can be assumed to be generated from the same SV event (**b3**). Next, all the SV signatures of a cluster are used to estimate the genomic coordinate (orange segment) of each SV event (**b4**). Finally, the number of SV signatures of a cluster and the total number of reads aligned in the breakpoints are used for genotyping with a maximum-likelihood Bayesian classification algorithm (**b5**). In panel (**c**) are reported the steps that GASOLINE follows for detecting somatic SVs. Somatic SVs are identified by comparing the SV signatures of a test (cancer) sample with a control (normal) sample. The SVs detected in the test sample (**c1**) are compared with the SVs signatures extracted from the control sample (**c2**) by calculating the NRO: SV signatures with a $NRO_{SV}$ larger than a predefined threshold are considered to be generated from the SV event of the test sample (**c3**). Statistical significance of each somatic SV is calculated by applying the Fisher’s exact test on the contingency table of (**c4**): $N_{RRT}$ (number of reads without SV signatures in test sample), $N_{VRT}$ (number of reads with the SV signatures in test sample), $N_{RRC}$ (number of reads without SV signatures in control sample), $N_{VRC}$ (number of reads with the SV signatures in control sample) . SVs with a p-value smaller than a predefined significance threshold are considered somatic.

To identify germline SVs, GASOLINE takes as input the aligned data of a sample (in BAM format) and extract the genomic coordinates of gap- and split-alignment signatures (Fig. 1a1 and Supplementary Fig. S1). SV signatures are then clustered with a sophisticated clustering procedure based on NRO measure, whose first step consists in calculating NRO for each pair of signatures (Fig. 1a2,a3).

Once $NRO_{ij}$ is calculated for all the signatures pairs [*i*, *j*] (Fig. 1b1), we perform interval clustering by using a graph-based approach. We first create an undirected graph by exploiting the NRO matrix as adjacency matrix, in which nodes are SV signatures and edges between two nodes *i* and *j* exist if $NRO_{ij}> Thr$ (where *Thr* is a predefined reciprocal overlap threshold, Fig. 1b2). An edge between two nodes expresses the confidence of two signatures being generated by the same SV event (Fig. 1b3).

The undirected graph is then used to extract maximal cliques (groups of fully connected nodes) by using the Eppstein–Löffler–Strash algorithm^21^. Maximal cliques represent groups of signatures that can be assumed to be generated from the same SV event (Fig. 1b3). SV signatures of each maximal clique are then used to estimate the genomic coordinates (start and end) of each SV event by calculating the median of all start and end coordinates (Fig. 1b4). For each cluster we then calculate different statistics including: cohesion score (the ratio between the numbers of links in the extended clique and the maximum numbers of link), mean mode and standard deviation of start and end coordinates. These statistics are then exploited to filter out low quality SV-signature clusters.

For deletions, duplications, inversions and insertions, the clustering procedure is applied separately to signatures extracted from each chromosome. Similarly, for translocations, clustering is applied to signatures extracted from each pair of chromosomes.

Finally, under the assumption of diploidy, each SV event is genotyped as reference, heterozygous, homozygous, by using the maximum-likelihood Bayesian classification algorithm as in^22^ (Fig. 1b5).

### Somatic GASOLINE

The detection of somatic SVs in cancer genomes consists in the identification of SVs present in the cancer sample and absent in the patient-matched normal sample. GASOLINE identifies somatic SVs by first applying the germline detection module to the test data and then searching for overlapping SVs signatures in the matched normal sample (Fig. 1c).

The signature clusters identified in the test sample are then compared with the signatures extracted from the control sample, by calculating their NRO (Fig. 1c2). Signatures with a $NRO_{SV}$ larger than a predefined threshold are considered to be generated from the SV event of the test sample (see Fig. 1c3).

Statistical significance of somatic SV is calculated by comparing the proportion between SV signatures and reference reads in the tumor and matched-normal samples with the Fisher’s exact test (contingency table of Fig. 1c4). SVs with a p-value smaller than a predefined significance threshold are considered somatic.

### GASOLINE and germline SV detection

Many computational methods have been developed to detect SVs from different technologies and evaluation of their performance is a very challenging task, mainly due to the lack of gold-standard datasets including all subtypes of structural variation.

Thus, to assess the performance of GASOLINE in the detection and genotyping of germline SVs we first generated synthetic genomes with SVs of all subtypes and size by using the PBSIM2 software^23^. PBSIM2 was exploited to simulate datasets mimicking the characteristics of long reads generated by either ONT or PacBio platforms, with average size of 10 kb, a global error rate of 90% and a total sequencing coverage from 5$\times$ to 30$\times$ (coverage = 5$\times$,10$\times$, 15$\times$, 20$\times$, 25$\times$ and 30$\times$).We simulated all SV subtypes (deletions, insertions, duplications, inversions and translocations) in homozygous and heterozygous state and with size ranging from 50 bp to 5 kb (50, 100, 200, 300, 400, 500, 1000, 2000, 3000, 4000, 5000 bp). Simulated sequencing datasets were then aligned to the reference genome with minimap2^24^ and NGMLR^18^ aligners (see “Methods”).

To investigate the performance of our tool as a function of the NRO threshold, we applied it to the synthetic dataset using different parameter settings (NRO=[0.5, 0.6, 0.7, 0.8, 0.9]) and calculated precision and recall as in^25^ (see “Methods”). The results of these analyses (Supplementary Figs. S2–S4) demonstrated that NRO thresholds have little effect on the global performance of our tool, with NGMLR-generated data giving better results with low NRO thresholds (0.5–0.7), minimap2-generated data requiring instead higher values (0.8–0.9), particularly in the case of deletions.

Recently, the Genome in a Bottle (GIAB) Consortium, using a combination of short-, linked-, and long-read sequencing, as well as optical mapping, has characterized the genome of an individual of Ashkenazim ancestry (NA24385), thus generating gold-standard datasets for SVs^26^. Although fundamental to test new technologies and algorithms, this dataset only contains high confidence sequence-resolved insertion and deletion calls $>50$ base pairs (bp), and it does not enable performance assessment for inversions, duplications and translocations.

For this reason, we simulated inversions, duplications and translocation by using a computational strategy based on SURVIVOR^27^ to modify the human reference genome and PBSIM2 to simulate ONT or PacBio reads (see “Methods”). Simulated reads were then aligned to the human reference genome with minimap2^24^ and NGMLR^18^ aligners (see “Methods”).

**Figure 2:**
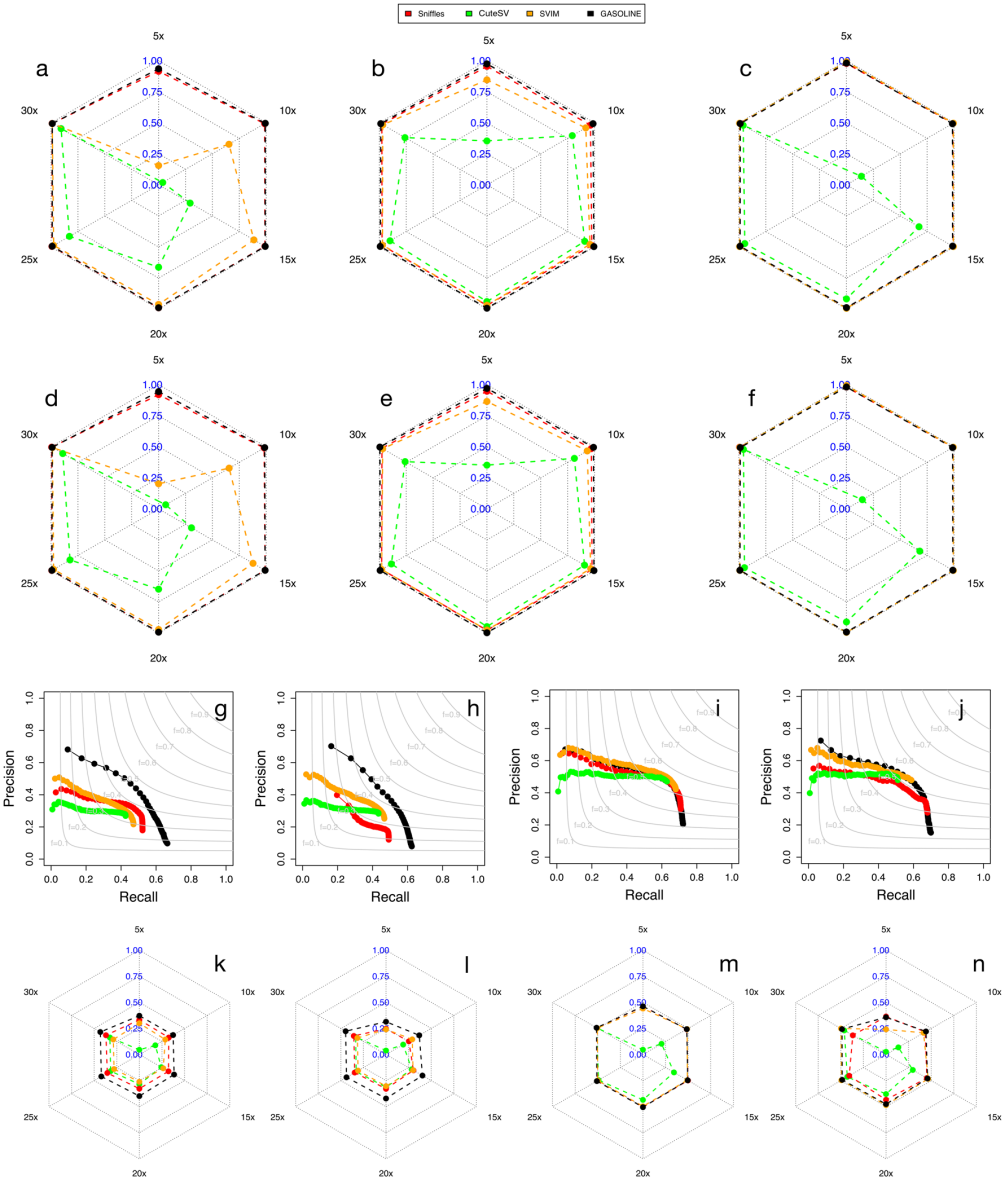
Global performance of GASOLINE and the other three tools in the detection of synthetic and real germline SVs. Panels a-f report the F1 score obtained by the four tools in the analysis of simulated inversions (**a**,**d**), duplications (**b**,**e**) and translocations (**c**,**f**). Results are reported for ONT (**a**–**c**) and PacBio (**d**,**e**) synthetic reads aligned with minimap2. Panels (**g**–**j**) report precision and recall obtained by the four tools in the analysis of the NA24385 datasets for the detection of small SVs (**g**,**h**) and large SVs (**i**,**j**) with ONT (**g**,**i**) and PacBio (**h**,**j**) data. The curves in panels (**g**–**j**) were obtained by ordering all the SVs as a function of number of supporting reads and calculating precision and recall including SVs with decreasing number of reads. Panels (**k**–**n**) show F1 score obtained by the four tools for the NA24385 datasets in the detection of small (**k**–**l**) and large SVs (**m**–**n**) with ONT (**k**,**m**) and PacBio (**l**,**n**) datasets at different sequencing coverages.

The synthetic dataset was then exploited to compare the performance of GASOLINE (using NRO=0.8) with those of other three state-of-the-art tools: Sniffles2^18^, CuteSV^17^ and SVIM^16^. The results reported in Fig. 2a–f and Supplementary Fig. S5 demonstrate that our method (and Sniffles2) obtained the best performance in the identification of simulated inversions, duplications and translocations, especially for low sequencing coverages (5–10$\times$), thus demonstrating that our new NRO-based computational strategy is capable to group SV signatures with an high level of accuracy.

Remarkably, sequencing coverage has little effects on the global performance of our tool for both alignment algorithms: as previously reported by^28^ a sequencing coverage higher than 15$\times$ is sufficient for detecting all subtypes of SVs and its increase does not lead to significant improvements.

To evaluate the accuracy of GASOLINE in the detection of real germline insertions and deletions we applied it to the publicly available ONT and PacBio NA24385 datasets generated by the GIAB consortium (see “Methods”) and compared its performance with the three aforementioned tools. To assess the performance of the four tools in detecting SVs at different sequencing coverages, we downsampled ONT and PacBio datasets to simulate 5, 10, 15, 20, 25 and 30$\times$ coverages and performed alignments with both minimap2 and NGMLR.

To compare the capability of the four tools to cluster SV signatures, we calculated precision and recall as a function of numbers of reads supporting each SV event, separately for large and small SVs (see “Methods”). Figure 2g–j and Supplementary Figs. S6–S17 show that for large insertions and deletions ($>500$ bp) all the tools performed very similarly. For small SVs ($<500$ bp), instead, GASOLINE showed the superiority of its NRO-based clustering procedure in grouping true SV signatures from long reads noisy alignments, obtaining the highest precision at the same level of recall for all sequencing coverages (5–30$\times$), sequencing data (PacBio and ONT) and aligners (NGMLR and minimap2). These analyses also showed that NGMLR alignment gave better results in the detection of large SVs, while minimap2 data better detected small SVs.

Finally, we calculated precision and recall for all SVs genotyped as heterozygous (0/1) or homozygous (1/1) by the four tools and we found that while for large insertions and deletions ($>500$ bp) all tools obtained very similar results, for small SVs ($<500$ bp) GASOLINE obtained the highest F1 measure for all sequencing coverages and technologies (Fig. 2k–n).

### Somatic SV detection on simulated and real data

As for germline variants, the validation of computational methods for somatic SVs detection is challenged by the lack of high-quality gold standard datasets enabling benchmarking and comparison of bioinformatic analysis pipelines, especially for tools exploiting long-read datasets and capable to identify small and complex variants previously unseen by short-reads WGS experiments.

Recently, Valle-Inclan et al.^19^, generated a comprehensive set of true somatic SVs (comprising all SV types) of the melanoma COLO829 cell lines by using four different sequencing technologies (Illumina HiSeq, ONT, PacBio and 10$\times$ Genomics) combined with extensive experimental validation (see “Methods”).

Despite the great utility of such a gold reference dataset, its application for benchmarking purposes on long read data is limited by the small number of insertions and by the size distribution of all the SVs that are mainly larger than 50 kb not allowing to evaluate the performance of algorithms in the detection of small SVs ([50, 500] bp).

For these reasons, to assess the accuracy of GASOLINE in the identification of small somatic insertions and deletions we simulated somatic SVs of different sizes by using the PacBio and ONT WGS NA24385 dataset generated by the GIAB consortium (see “Methods”). We selected 330 heterozygous SVs (169 insertions and 161 deletions from the high-confidence callsets generated by the GIAB consortium) with size distribution in the range [50 bp, 5 kb] (with 230 SVs smaller than 1 kb). Using custom scripts, we first removed all reads containing signatures of the 330 SVs, we randomly splitted the remaining reads in two sets and finally, we added all the reads containing SV signatures to one of the splitted sets. With this workflow, for both PacBio and ONT data we obtained a $\sim 30\times$ sequencing experiment with reads containing the 330 selected SVs (test), and a $\sim 30\times$ control experiment without the 330 SVs. To evaluate detection accuracy at different sequencing coverages we downsampled read datasets to obtain 5$\times$, 10$\times$, 15$\times$, 20$\times$ and 25$\times$. Raw reads were aligned using either minimap2 or NGMLR. We then applied the somatic module of GASOLINE to the simulated datasets and we compared its performance to the other three tools (see “Methods”). Sniffles2, SVIM and CuteSV were used as in Valle-Inclan et al.^19^. They were applied separately on test and control samples and somatic SVs were called discarding events with supporting reads in the control sample. Supporting signatures in control samples were searched by using a reciprocal overlap larger than 0.5.

**Figure 3:**
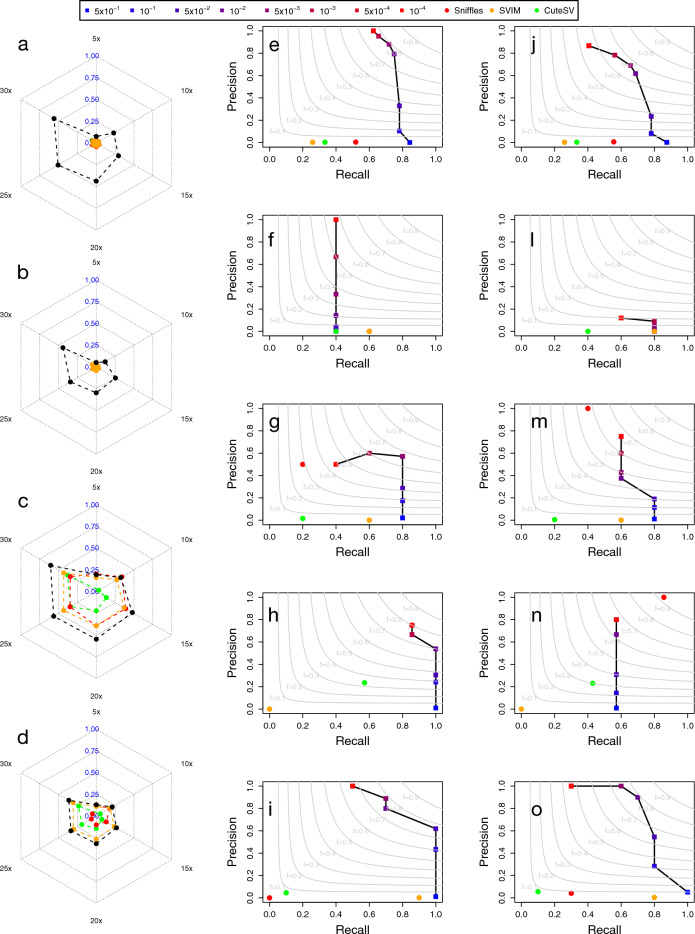
Performance of GASOLINE on the detection of somatic SVs. Panels (**a**–**d**) show F1 score obtained by the four tools in the detection of simulated small (**a**,**b**) and large somatic SVs (**c**,**d**) with ONT (**a**,**c**) and PacBio (**b**,**d**) datasets at different sequencing coverage. Panels (**e**–**o**) report the precision-recall obtained by GASOLINE and the other three tools in the detection of deletions (**e**,**j**), insertions (**f**,**l**), duplications (**g**,**m**), inversions (**h**,**n**) and translocations (**i**,**o**) of the Valle-Inclan et al. true-set for the COLO829 cell lines sequenced with ONT (**e**–**i**) and PacBio (**j**–**o**) technologies. The results for GASOLINE were reported for different somatic p-value thresholds ($5 \times 10^{-1}$, $1 \times 10^{-1}$, $5 \times 10^{-2}$, $1 \times 10^{-2}$, $5 \times 10^{-3}$, $1 \times 10^{-3}$, $5 \times 10^{-4}$, $1 \times 10^{-4}$). All the results reported in the panels are based on mimimap2 alignment data.

GASOLINE obtained the best F-measure for all sequencing coverages of both PacBio and ONT datasets aligned with minimap2 or NGMLR (Fig. 3a–d and Supplementary Figs. S18–S29), especially in the detection of small SVs ($[50{-}500]$). In contrast, the other three tools identified a large number of false positive somatic SVs resulting in very low levels of precision. These analyses also showed that low sequencing coverages ($\le 15x$) yield very poor performance and that, similarly to germline SVs, the best F1-measure is obtained with at least 20$\times$ coverage, for both PacBio and ONT data. Notably, ONT data outperformed PacBio in all our analyses, while the minimap2 approach showed higher precision and recall than obtained with NGMLR.

We next tested the capability of our tool in detecting all SVs subtypes, applying it to the ONT and PacBio data of the COLO829 cell lines and comparing its performance with those of the other three tools by using the Valle-Inclan et al.^19^ true-set as benchmark (see “Methods”). As with the simulated datasets, the three state of the art tools identified a large number of false positive somatic events that generated very low levels of precision. On the other hands, GASOLINE, filtering out SVs on the basis of somatic p-values, was capable to drastically increase precision (removing a large fraction of false positive calls) at the expense of a minimal decrease in recall (removal of true positive calls). For all SV subtypes, with the exception of insertions (unfortunately the gold standard true dataset only contains three somatic insertions), our method was capable to identify between 80% and 100% of the Valle-Inclan et al. true-set with precision in the order of 60–80%, outperforming the other three state of the art methods (Fig. 3e–o).

Notably, in ONT analyses with somatic p-value< 0.001, GASOLINE detected 49 SVs (25 deletions, 4 insertions, 9 Inversions, 5 tandem duplications and 6 translocations). Among these variants, 6 SVs (1 deletion, 1 insertions, 1 duplication and 3 inversions) were not present in the Valle-Inclan et al. gold reference set (Supplementary Table S1) and were not detected by the other three tools. Visual inspection of aligned reads demonstrated that all the six SVs have somatic signatures in ONT data (signatures in cancer and not in normal). Moreover, 5 out 6 of these SVs are also supported by Pacbio and/or Illumina data of the COLO829 cell line (see Supplementary Figs. S30–S35).These results demonstrate that GASOLINE can expand our potential to study somatic alterations in cancer and that the precision and recall reported in Fig. 3 are even underestimated.

### GASOLINE tool

GASOLINE is a collection of Perl, R and Fortran codes for the detection of somatic SVs from long read sequencing data. It takes as input two BAM files from a pair of test and matched normal samples and gives as output a VCF file (version 4.2) with statistically significant somatic SVs.

GASOLINE analyzes aligned data in BAM format and extracts the genomic coordinates of discordant alignments of a read with respect to the reference genome (SV signatures). The parsing module of our tool searches for two types of signatures: gapped alignments (in the CIGAR strings) and split alignments (primary and supplementary alignment of a read).

At present the signature extraction module is written in Perl and takes around 3 h for parsing a ONT or PacBio WGS at 30$\times$ of sequencing coverage. After the parsing step, GASOLINE can be run in ‘germline’ or ‘somatic’ mode. In ‘germline’ mode, SV signatures are grouped with the NRO-based clustering and then genotyped. In ‘somatic’ mode SV signatures from a test cancer sample are clustered and then compared with those of matching control sample to calculate somatic p-value with Fisher’s exact test.

Both ‘germline’ and ‘somatic’ modules can be ran in multicores: the ‘germline’ module takes around 1 h to genotype a 30$\times$ bam file (with 20 threads), while the ‘somatic’ module takes around 2 h two compare the SV signatures of two 30$\times$ bam files (with 20 threads).

GASOLINE can run on any UNIX system (desktops and workstations). The GASOLINE tool is freely available at https://sourceforge.net/projects/gasoline/.

## Discussion and conclusion

Long-read sequencing technologies are revolutionizing our capability of identifying and resolve the structure of complex SVs with an unprecedented accuracy and resolution. However, currently available tools for long-read analyses are based on computational procedures that limit the detection of small SVs ($<500$ bp). Notably, none of them is properly devised for the identification of somatic alterations.

In order to overcome the limits of currently available methods, we developed GASOLINE, the first computational approach that is capable of detecting germline and somatic SVs from long reads sequencing datasets. GASOLINE is based on a novel reciprocal overlap (NRO) criterion that allows to group both small and large SV signatures with high accuracy, thus reducing the effect of imprecise alignment and allowing the identification of both small ($<500$ bp) and large ($>500$ bp) SVs with the same accuracy.

Analyses of synthetic and real long-read datasets demonstrated that our NRO-based clustering algorithm clearly outperform the other state of the art method in the detection of germline alterations, especially SVs smaller than 500 bp.

The most important novelty and uniqueness of GASOLINE lies in its capability to compare a test and a matched normal sample to identify somatic alterations. At present, the standard approach for the detection of somatic variants consists in applying these methods separately on paired samples (test and control) discarding SVs with a supporting read in the control sample. GASOLINE directly compares the SV signatures found in test and control samples and then it calculates somatic statistical significance with Fisher’s exact test.

As for germline variants we tested the performance of our tool in the detection somatic variants, using both simulated and real long-read cancer datasets. In synthetic datasets, as for germline variants, our tool demonstrated its superiority in the detection of small SVs for all sequencing coverages we simulated. In particular, the somatic p-values calculated by GASOLINE are a useful instrument to increase precision (removing a large fraction of false positive calls) at the expense of a minimal decrease in recall (removal of true positive calls).

When applied on a pair of metastatic cutaneous melanoma (COLO829) and matched normal sample, GASOLINE outperformed the other three tools in the detection of all SV subtypes. Notably, our tool identified five genuine somatic SVs that were missed by Valle-Inclan et al. by using five different sequencing technologies and state-of-the art SV calling approaches, demonstrating that GASOLINE can expand our capability of studying somatic alterations in cancer.

At present, the speed of GASOLINE is, however, still slower than the other of state-of-the-art approaches. The germline or somatic analysis of a 30$\times$ coverage sequencing experiments requires 4–5 h with 20 threads. This is mainly due to the SV signature extraction module of our method that is implemented in perl. We are planning to implement the parsing module in c++, to obtain computational speed comparable to that of currently available state of the art SV callers.

Regardless, the results obtained in all of the comparative analyses we performed highlighted the versatility of our software and its ability to overcome the limitations and drawbacks of currently available state-of-the-art tools, thus making GASOLINE a suitable tool for the investigation of SVs in population as well as in cancer studies.

## Materials and methods

### PBSIM2

PBSIM2 simulates synthetic reads by randomly sampling from a reference sequence, adding errors with user defined distribution of substitutions, insertions and deletions and allowing to define read size distribution (mean and maximum size) and the desired sequencing coverage. PBSIM2 was exploited to simulate sequencing dataset that mimic the characteristics of long reads generated by ONT and PacBio platforms with total sequencing coverage from 5$\times$ to 30$\times$ (coverage = 5$\times$, 10$\times$, 15$\times$, 20$\times$, 25$\times$ and 30$\times$). To study the performance of GASOLINE as a function of NRO values, we simulated all SV subtypes in homozygous and heterozygous state by applying PBSIM2 to reference sequences obtained by modifying a 5 Mb segment of chromosome 1 of the hg19 (1:5000001–10000000, see “Methods”) with deleted, inserted, duplicated and inverted segments ranging from 50 bp to 5 kb (50 bp, 100 bp, 200 bp,...). Translocations were simulated by applying PBSIM2 to two reference sequences obtained by modifying two 5 Mb segment of chromosome 1 of the hg19 (1:5000001–10000000 and 1:10000001–15000000, see “Methods”). Simulated reads were then aligned to the 5 Mb reference genomes with minimap2 and NGMLR aligners. To compare the performance of GASOLINE with other three state of the art methods, we simulated inversions, duplications and translocations by combining SURVIVOR (https://github.com/fritzsedlazeck/SURVIVOR) and PBSIM2. SURVIVOR was used to generate a modified version of the Human Genome (hg19) with 3000 inversions, duplications and translocations ranging from 500 bp to 30 kb. The modified and standard human genome was used to generate ONT and PacBio reads at 2.5$\times$, 5$\times$, 7.5$\times$, 10$\times$, 12.5$\times$ and 15$\times$ with PBSIM2. Reads from standard and modified genome were combined to obtain heterozygous SVs and aligned with minimap2 and NGMLR.

### Tools comparison

We downloaded the cuteSV tool (version 1.0.12) from https://github.com/tjiangHIT/cuteSV, Sniffles2 (version 2.0.7) from https://github.com/fritzsedlazeck/Sniffles and SVIM from https://github.com/eldariont/svim. For germline analyses, CuteSV was applied by using parameter settings suggested in the github page for ONT data (–max_cluster_bias_INS=100, –diff_ratio_merging_INS=0.3, –max_cluster_bias_DEL=100, –diff_ratio_merging_DEL=0.3) and for PacBio data (–max_cluster_bias_INS=100, –diff_ratio_merging_INS=0.3, –max_cluster_bias_DEL=200, –diff_ratio_merging_DEL=0.5), while Sniffles2 and SVIM were both run with default parameter settings.

Somatic analyses were performed by using each tool separately on paired samples (test and control) and discarding SVs with a supporting signature in the control sample. Supporting signatures in control samples were searched by using a reciprocal overlap larger than 0.5. GASOLINE was applied to all the datasets (PBSIM2 synthetic datasets, the NA24385 datasets, the COLO829 datasets and the synthetic somatic SVs generated with the NA24385 dataset), in ‘germline’ and ‘somatic’ mode with $NRO=0.8$, $NO_{Norm}=1000$. In all the analyses we performed, precision was calculated as the ratio between the number of correctly detected events (the intersection between the tool calls and the gold standard set calls) and the total number of events detected by each method, while recall was calculated as the ratio between the number of correctly detected events and the total number of events in the gold standard dataset (as in^25^). In both germline and somatic analyses each SV was considered a true positive if we found a reciprocal overlap larger than or equal to 50% with an SV of the validation set. SVs detected by GASOLINE on the COLO829 datasets with p-value$<0.001$ were validated by visual inspection by using the Integrative Genomics Viewer^29^ (IGV version 2.9.4, https://software.broadinstitute.org/software/igv/download) and Samplot (version 1.3.0, https://github.com/ryanlayer/samplot)^30^.

### NA24385 data

ONT and PacBio read data for the NA24385 individual of Ashkenazim ancestry was obtained from the GIAB ftp site https://ftp-trace.ncbi.nlm.nih.gov/giab/ftp/data/AshkenazimTrio/HG002_NA24385_son/. Reads in fastq (for ONT) or fasta (for PacBio) format were aligned against the human reference genome (hg19) by using minimap2 and NGMLR aligners, obtaining an average coverage of 64$\times$ for ONT and Xx for PacBio. To simulate 5, 10, 15, 20, 25 and 30$\times$ sequencing coverages, the original reads in fastq and fasta formats were downsampled by using the seqtk tool (https://github.com/lh3/seqtk) and then aligned to the human reference genome (hg19) with minimap2 and NGMLR. The GIAB consortium, by combining short-, long-, linked-read sequencing and optical mapping generated a high-quality callset of germline insertions and deletions. The NA24385 truth SV callset contains 12,745 SVs divided into 7,281 (6341 smaller than 1 kb and 787 in the range [1 kb, 5 kb]) insertions and 5,464 (4846 smaller than 1 kb and 462 in the range [1 kb, 5 kb]) deletions. The truth SV callset was downloaded at https://ftp-trace.ncbi.nlm.nih.gov/ReferenceSamples/giab/data/AshkenazimTrio/analysis/NIST_SVs_Integration_v0.6/.

### COLO829 data

The COLO829 cancer cell-line from a metastatic cutaneous melanoma patient and the COLO829BL cell-line from a lymphoblastoid line of the same patient were recently sequenced by Valle-Inclan et al.^19^, by using five different technology platforms (Illumina HiSeq Xten, ONT, PacBio, 10$\times$ genomics and Bionano Genomics Saphyr optical mapping). By using state-of-the art SV calling approaches generated a somatic SV truth set comprising 68 high confidence calls: 38 deletions, 3 insertions, 7 duplications, 7 inversions and 13 translocations. The somatic SV truth set was downloaded from 10.5281/zenodo.3988185. ONT, Illumina and PacBio WGS data were downloaded from EGA project PRJEB27698 (https://www.ebi.ac.uk/ena/browser/view/PRJEB27698). ONT and PacBio reads in fastq format were aligned against the human reference genome (hg19) by using minimap2 aligner, obtaining an average coverage of 60$\times$ for ONT and 50$\times$ for PacBio. Illumina reads in fastq format were aligned against the human reference genome (hg19) by using the Burrows-Wheeler Aligner (BWA)^31^, followed by indel realignment with GATK^32^.

### Supplementary Information

Supplementary Information.

## Data Availability

ONT and PacBio read data for the NA24385 individual of Ashkenazim ancestry was obtained from the GIAB ftp site https://ftp-trace.ncbi.nlm.nih.gov/giab/ftp/data/AshkenazimTrio/HG002_NA24385_son/. For COLO829 dataset, the somatic SV truth set was downloaded from https://zenodo.org/records/3988185. ONT, Illumina and PacBio WGS data were downloaded from EGA project PRJEB27698 (https://www.ebi.ac.uk/ena/browser/view/PRJEB27698).
