# Supplementary material for: GASOLINE: detecting germline and somatic structural variants from long-reads data
Source: Sci Rep. 2023 Nov 27;13:20817. doi: 10.1038/s41598-023-48285-0 (PMC10682169; doi:10.1038/s41598-023-48285-0)
Supplement: Supplementary file 1 — Supplementary Information. [file 41598_2023_48285_MOESM1_ESM.pdf]

Supplemental Methods to "GASOLINE: detecting germline and somatic structural variants from long-reads data."

Alberto Magi[1,2,+,*], Gianluca Mattei[1,+], Alessandra Mingrino[3], Chiara Caprioli[4,5], Chiara Ronchini[4], Gianmaria Frigè[4,5], Roberto Semeraro[3], Marta Baragli[1], Davide Bolognini[3], Emanuela Colombo[4], Luca Mazzarella[4], Pier Giuseppe Pelicci[4,5,*].

[1]Department of Information Engineering, University of Florence, Florence, Italy, albertomagi@gmail.com. [2]Institute for Biomedical Technologies, National Research Council, Segrate, Milano, Italy. [3]Department of Experimental and Clinical Medicine, University of Florence, Florence, Italy. [4]Department of Experimental Oncology, IEO European Institute of Oncology IRCCS, Milano, Italy. [5]Department of Oncology and Hemato-Oncology, University of Milan, Milan, Italy. [*] **Correspondence**: Alberto Magi, Department of Information Engineering, University of Florence, 50100, Florence, Italy (albertomagi@gmail.com), Pier Giuseppe Pelicci, Department of Experimental Oncology, IEO European Institute of Oncology IRCCS, Milano, Italy (piergiuseppe.pelicci@ieo.it) . [+] These authors contributed equally to this work.

Supplementary Table S1: Visual inspection of somatic SVs detected by GASOLINE (p-value$\leq$ 0.001) on COLO829 cell line and not present in the Valle-Inclan et al. true-set.

Chr	Start	End	Size	Type	TS	CS	P-value	ONT	PacBio	Illumina	Other	TP	Visualization
4	187996371	187996371	52	Ins	(13/62)	(0/46)	$1x10^{-3}$	Somatic	Somatic	X	No	TP	IGV
15	23712619	23714040	1421	Del	(17/101)	(0/56)	$9x10^{-4}$	Somatic	Somatic	Not Present	No	TP	Samplot
12	69629947	69630523	576	Dup	(16/41)	(1/55)	$6x10^{-5}$	Somatic	Not Present	Not Present	No	FP	Samplot
7	126167444	143939814	17772370	Inv	(15/86)	(0/60)	$6x10^{-4}$	Somatic	Somatic	No Somatic	No	TP	Samplot
7	143938346	144090465	152119	Inv	(24/96)	(0/47)	$1x10^{-4}$	Somatic	Somatic	Not Present	No	TP	Samplot
7	143936534	143937206	672	Inv	(24/94)	(0/46)	$1x10^{-4}$	Somatic	Somatic	Somatic	No	TP	Samplot

Columns report the genomic coordinates (Chr, Start and End, with respect to hg19), the size (in bp), the type of SV (Del, Ins, Dup, Inv), the number of signatures supporting the SV and the total number of reads with reference allele in the cancer sample (TS, signature number are separated by /), the number of signatures supporting the SV and the total number of reads with reference allele in the control sample (CS, signature number are separated by /), the somatic p-value calculated by GASOLINE (), the status of somatic variant in ONT data (evaluated by visual inspection of SV signatures present in tumor sample and in control sample. The variant is considered somatic if the number of signatures in control sample is at most one), the somatic status of the SV in Pacific Bioscience and Illumina sequencing data (evaluated as for ONT data). The last four columns report if the SV was detected by the other three tools (Other), if the SV has been considered a novel true positive (TP) based on signatures of PacBio and Illumina data and the visualization tool used for visual inspection (Samplot or IGV).

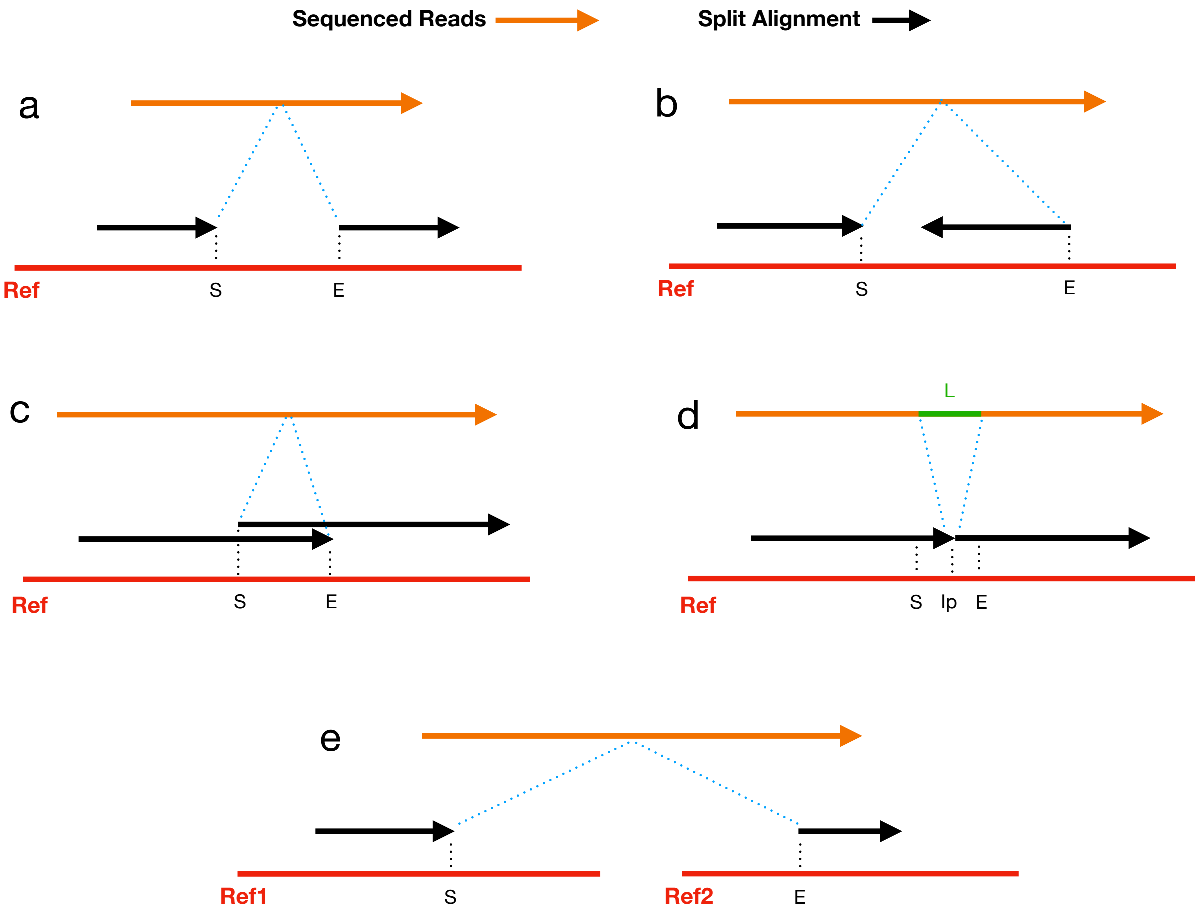

Supplementary Figure S1: Schematic illustration of split read SV signatures. Panels show how SVs generate typical split read alignment signatures. Two consecutive segments mapping far apart with the same orientation define a deletions signature (a). Two consecutive segments mapping far apart with opposite orientation define an inversion signature (b). When two consecutive segments have overlapping coordinates they define a duplication signature (c). A read splitted in three segments, with the first and third segments closely mapped, define an insertion signature (d). Finally, when two consecutive segments mapped on different chromosomes define a translocation signature (e). For each split alignment S (Start) and E (End) represent the genomic coordinates of the signature. For deletions, inversions and translocations, S is defined as the last mapping position of the first segment, while E is the first mapping position of the second segment. For duplications, S is the first mapping position of the second segment, while E is the last mapping position of the first segment. Since insertions are defined by only one coordinate (Ip), we consider S=Ip-L/2 and E=Ip+L/2, where L is the size of the inserted segment.

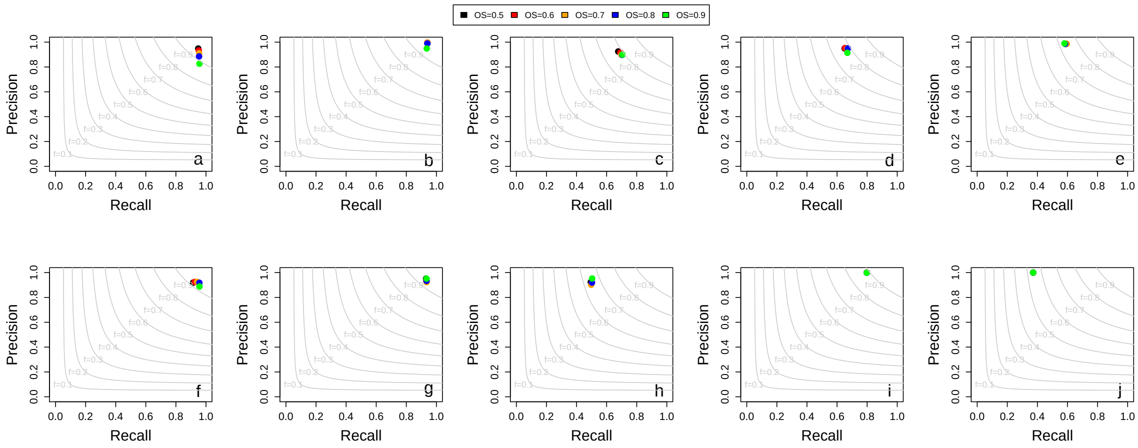

Supplementary Figure S2: Global performance of GASOLINE and the other three tools on synthetic data. Panels a-e report the precision-recall plots for synthetic reads aligned with NGMLR, while panels f-j for synthetic reads aligned with minimap2. Panels a and f reports the performance of the four methods in the detection of synthetic deletions, panels b and g for synthetic insertions, panels c and h for duplications, d and i for inversions and e and j for translocations.

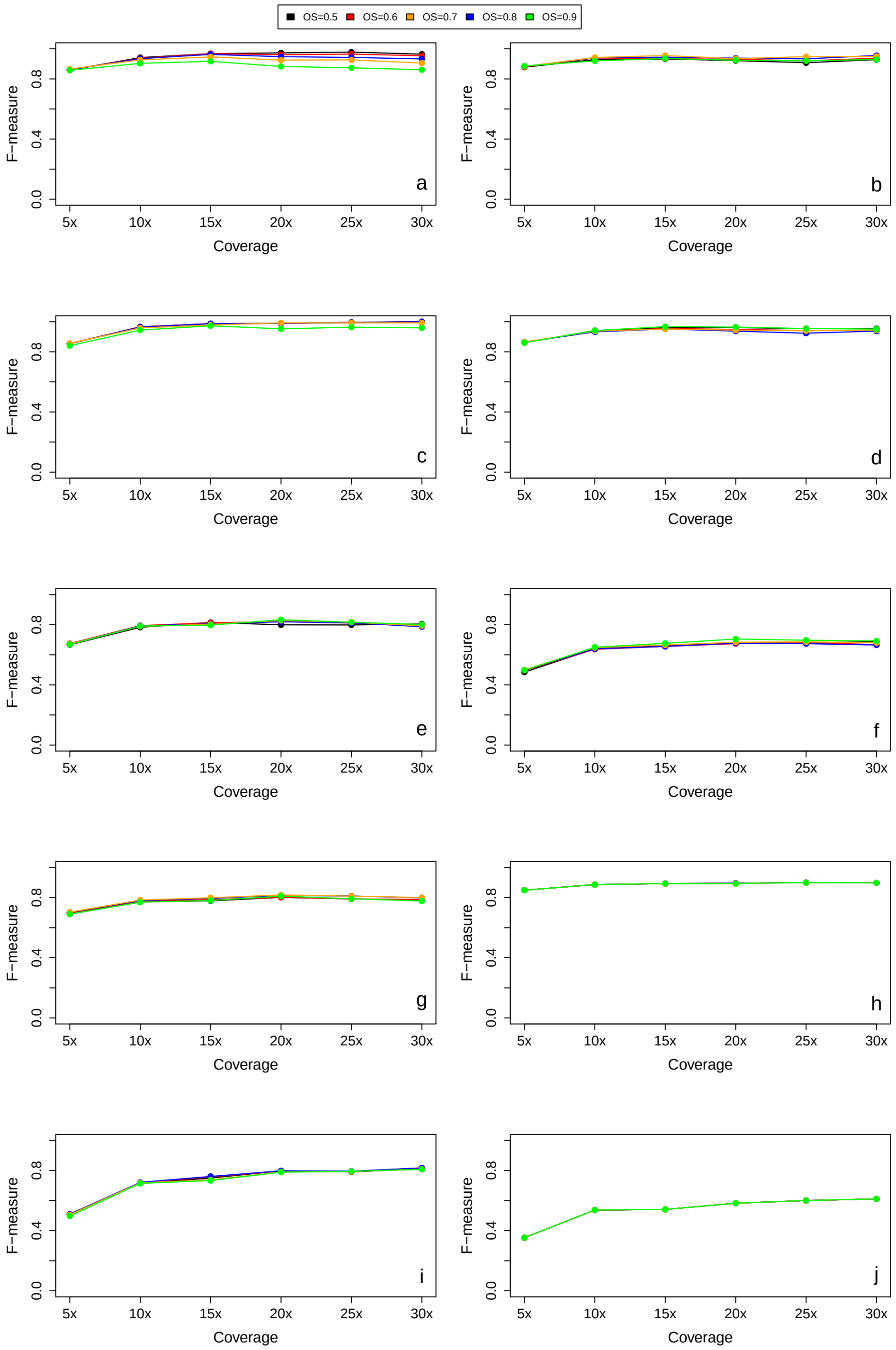

Supplementary Figure S3: Performance of GASOLINE with different NRO values on synthetic data as a function of sequencing coverage. Figure reports the armonic mean of precision and recall (F-measure) obtained by GASOLINE in the detection of deletions (panels a and b), insertions (panels c and d), duplications (panels e and f), inversions (panels g and h) and translocations (panels i and j) from simulated data with different sequencing covarages (5x, 10x, 15x, 20x, 25x, 30x). Result are reported for sequencing data aligned with minimap2 (panels a, c, e, g, i) and NGMLR (panels b, d, f, h, j). GASOLINE was tested for different NRO values thresholds (NRO=0.5, NRO=0.6, NRO=0.7, NRO=0.8, NRO=0.9).

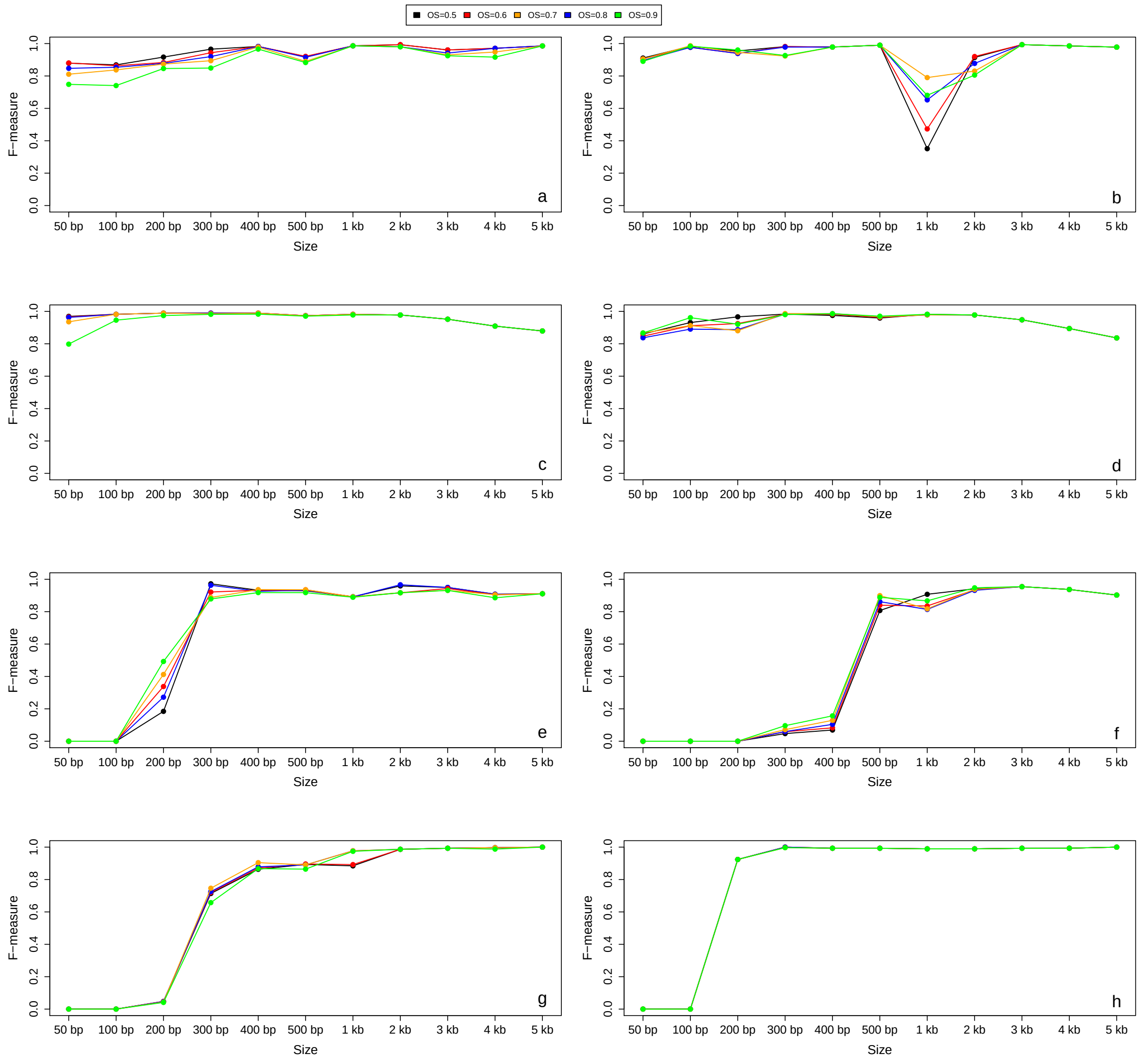

Supplementary Figure S4: Performance of GASOLINE with different NRO values on synthetic data as a function of SV size. Figure reports the armonic mean of precision and recall (F-measure) obtained by GASOLINE in the detection of deletions (panels a and b), insertions (panels c and d), duplications (panels e and f), inversions (panels g and h) and translocations (panels i and j) of different size (50 bp, 100 bp, 200 bp, 300 bo, 400 bp, 500 bp, 1 kb, 2 kb, 3 kb, 4 kb, 5 kb). Result are reported for sequencing data aligned with minimap2 (panels a, c, e, g, i) and NGMLR (panels b, d, f, h, j). GASOLINE was tested for different NRO values thresholds (NRO=0.5, NRO=0.6, NRO=0.7, NRO=0.8, NRO=0.9).

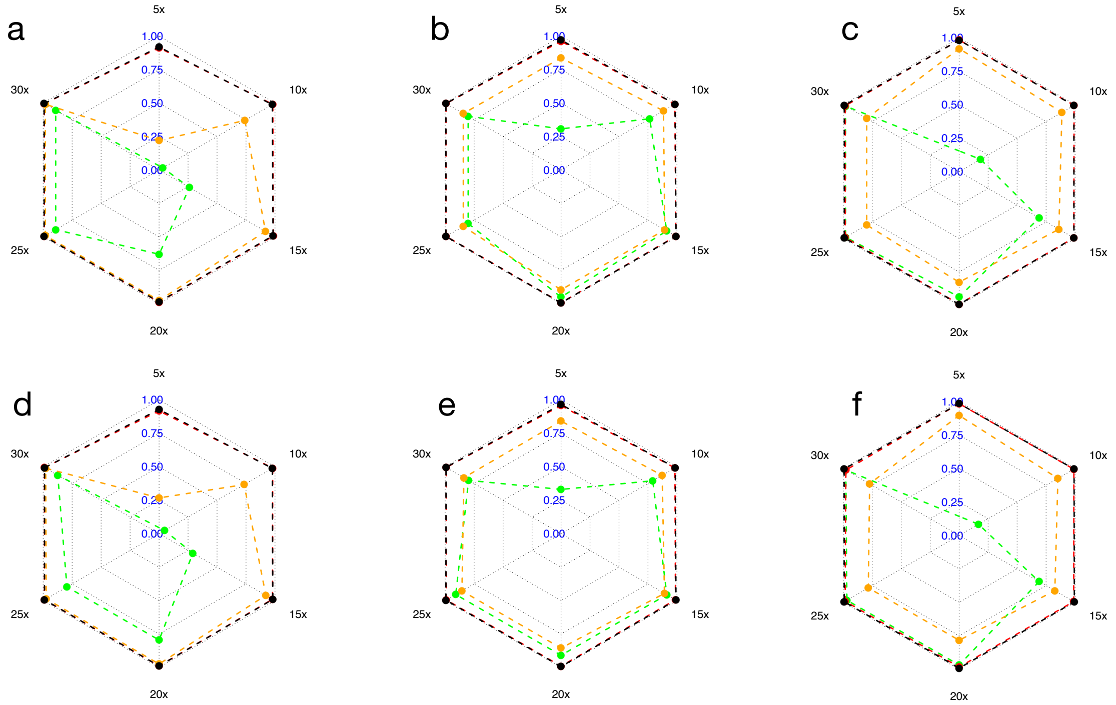

Supplementary Figure S5: Panels a-f report the F1 score obtained by the four tools in the analysis of simulated inversions (a,d), duplications (b,e) and translocations (c, f). Results are reported for ONT (a-c) and PacBio (d-e) synthetic reads aligned with NGMLR.

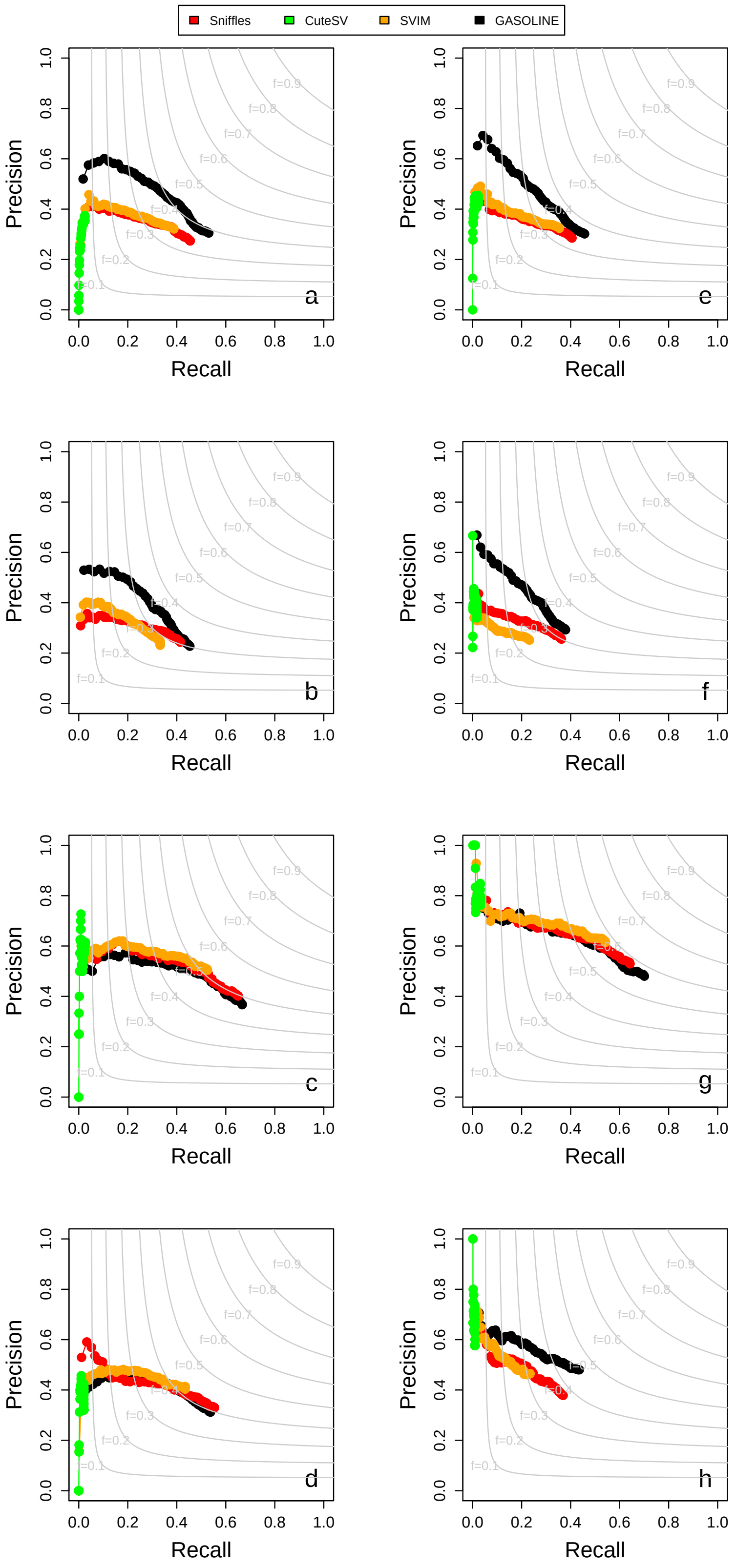

Supplementary Figure S6: Precision-Recall curves of GASOLINE and the other three tools as a function of number of supporting reads for NA24385 ONT data downsampled at 5x. Panels (a-d) show the results for minimap2 alignment and (e-h) for NGMLR. Panels (a, e) for small deletions (< 500 bp), (b, f) for small insertions (< 500 bp), (a, e) for large deletions (> 500 bp), (a, e) for large insertions (> 500 bp). The curves in panels were obtained by ordering all the SVs as a function of number of supporting reads and calculating precision and recall including SVs with decreasing number of reads.

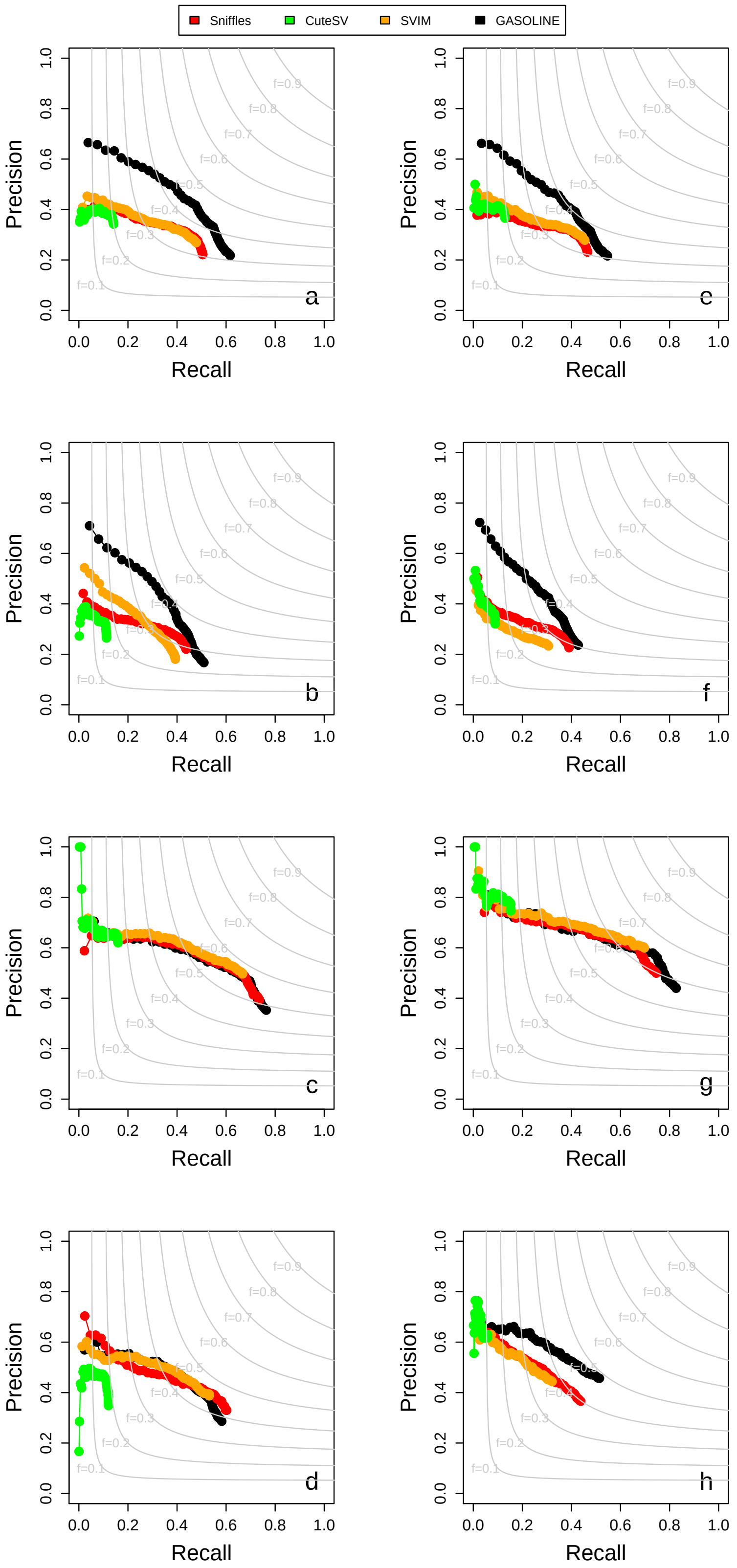

Supplementary Figure S7: Precision-Recall curves of GASOLINE and the other three tools as a function of number of supporting reads for NA24385 ONT data downsampled at 10x. Panels (a-d) show the results for minimap2 alignment and (e-h) for NGMLR. Panels (a, e) for small deletions (< 500 bp), (b, f) for small insertions (< 500 bp), (a, e) for large deletions (> 500 bp), (a, e) for large insertions (> 500 bp). The curves in panels were obtained by ordering all the SVs as a function of number of supporting reads and calculating precision and recall including SVs with decreasing number of reads.

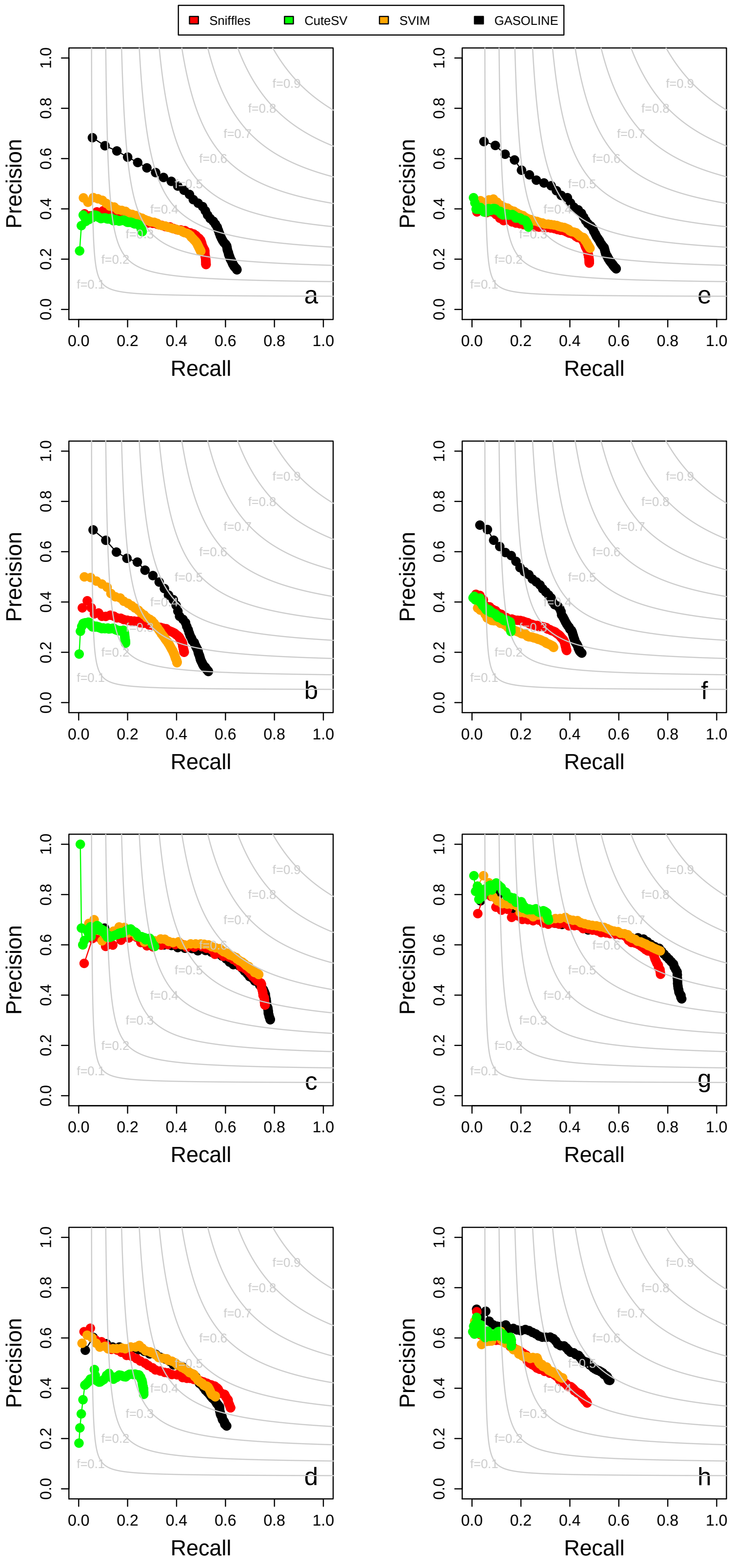

Supplementary Figure S8: Precision-Recall curves of GASOLINE and the other three tools as a function of number of supporting reads for NA24385 ONT data downsampled at 15x. Panels (a-d) show the results for minimap2 alignment and (e-h) for NGMLR. Panels (a, e) for small deletions (< 500 bp), (b, f) for small insertions (< 500 bp), (a, e) for large deletions (> 500 bp), (a, e) for large insertions (> 500 bp). The curves in panels were obtained by ordering all the SVs as a function of number of supporting reads and calculating precision and recall including SVs with decreasing number of reads.

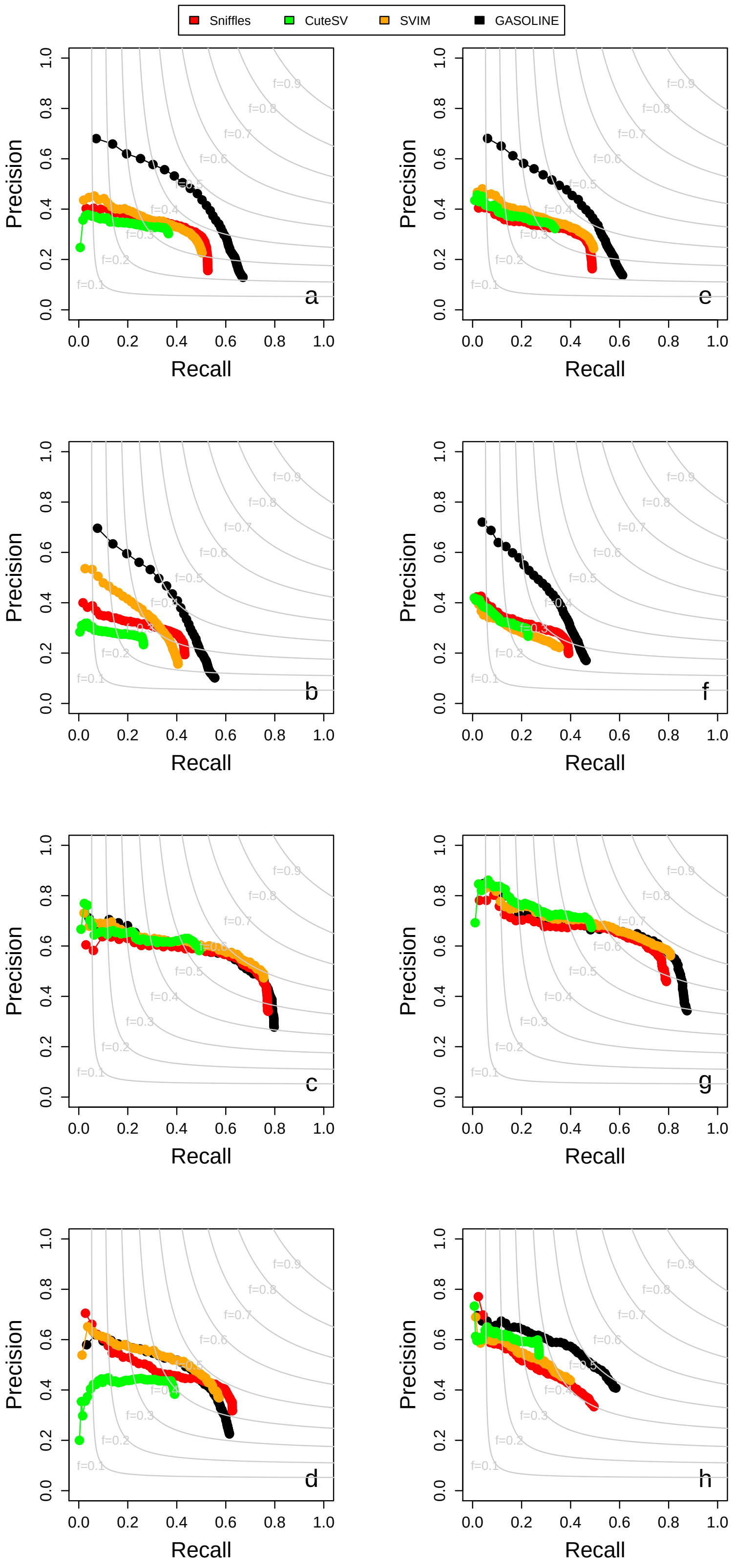

Supplementary Figure S9: Precision-Recall curves of GASOLINE and the other three tools as a function of number of supporting reads for NA24385 ONT data downsampled at 20x. Panels (a-d) show the results for minimap2 alignment and (e-h) for NGMLR. Panels (a, e) for small deletions (< 500 bp), (b, f) for small insertions (< 500 bp), (a, e) for large deletions (> 500 bp), (a, e) for large insertions (> 500 bp). The curves in panels were obtained by ordering all the SVs as a function of number of supporting reads and calculating precision and recall including SVs with decreasing number of reads.

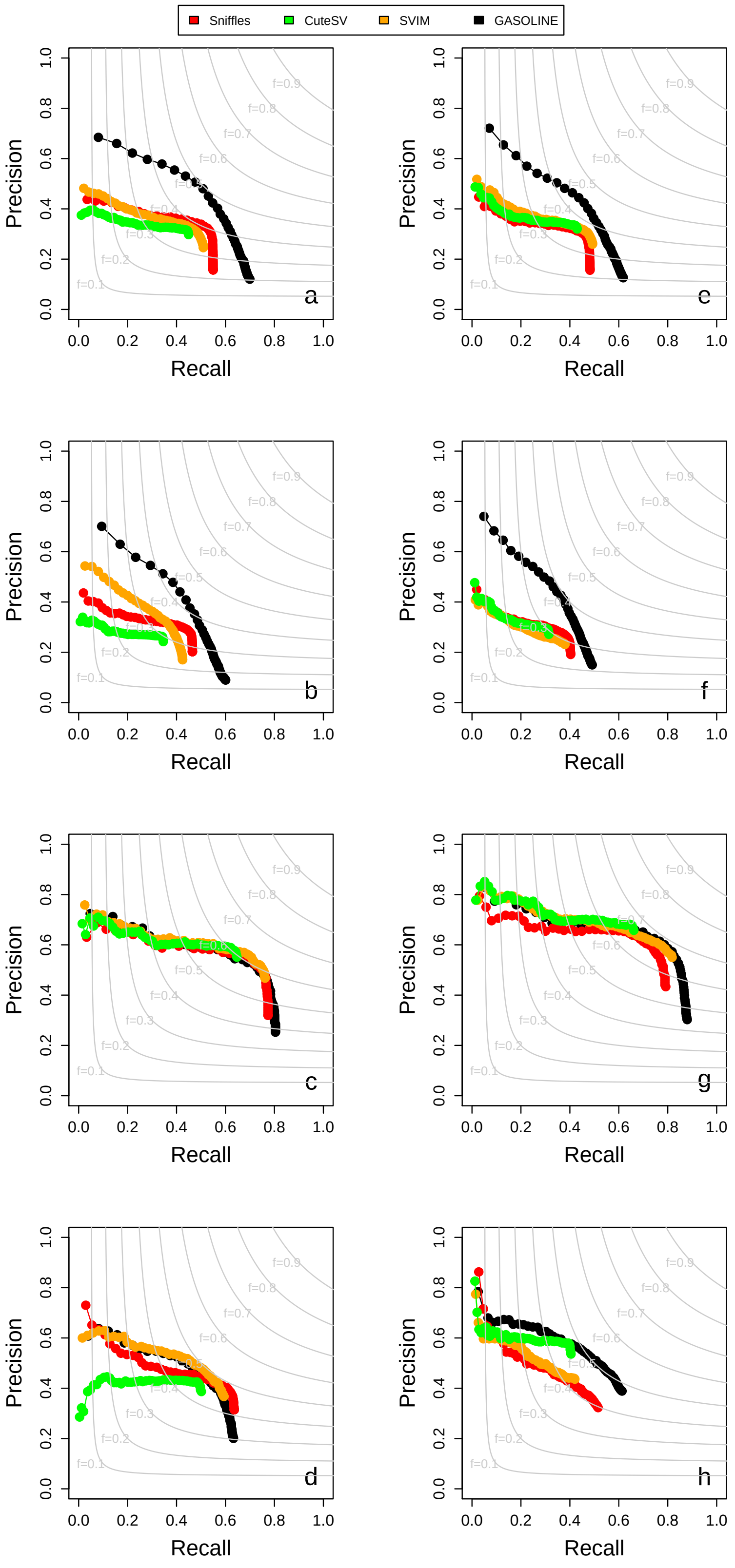

Supplementary Figure S10: Precision-Recall curves of GASOLINE and the other three tools as a function of number of supporting reads for NA24385 ONT data downsampled at 25x. Panels (a-d) show the results for minimap2 alignment and (e-h) for NGMLR. Panels (a, e) for small deletions (< 500 bp), (b, f) for small insertions (< 500 bp), (a, e) for large deletions (> 500 bp), (a, e) for large insertions (> 500 bp). The curves in panels were obtained by ordering all the SVs as a function of number of supporting reads and calculating precision and recall including SVs with decreasing number of reads.

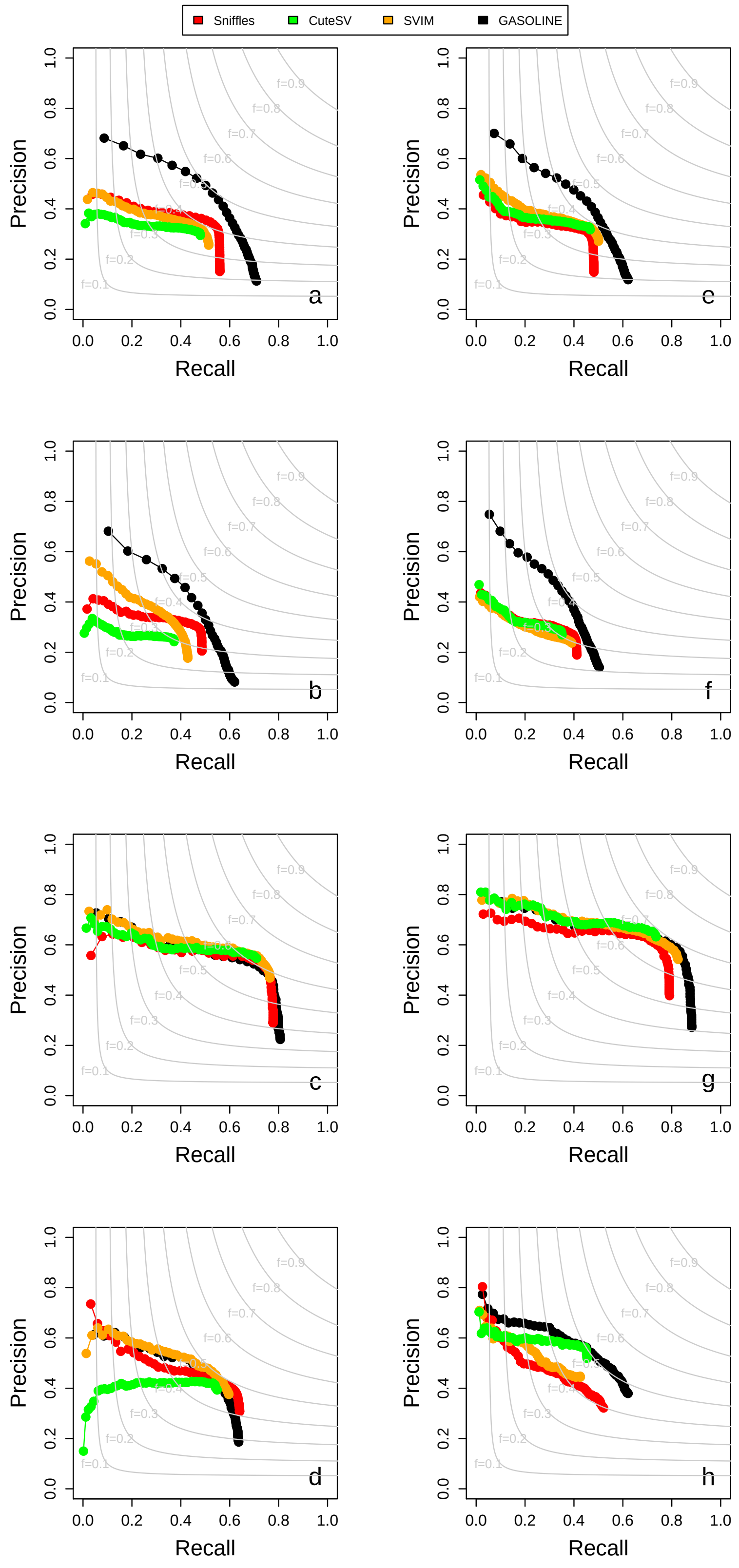

Supplementary Figure S11: Precision-Recall curves of GASOLINE and the other three tools as a function of number of supporting reads for NA24385 ONT data downsampled at 30x. Panels (a-d) show the results for minimap2 alignment and (e-h) for NGMLR. Panels (a, e) for small deletions (< 500 bp), (b, f) for small insertions (< 500 bp), (a, e) for large deletions (> 500 bp), (a, e) for large insertions (> 500 bp). The curves in panels were obtained by ordering all the SVs as a function of number of supporting reads and calculating precision and recall including SVs with decreasing number of reads.

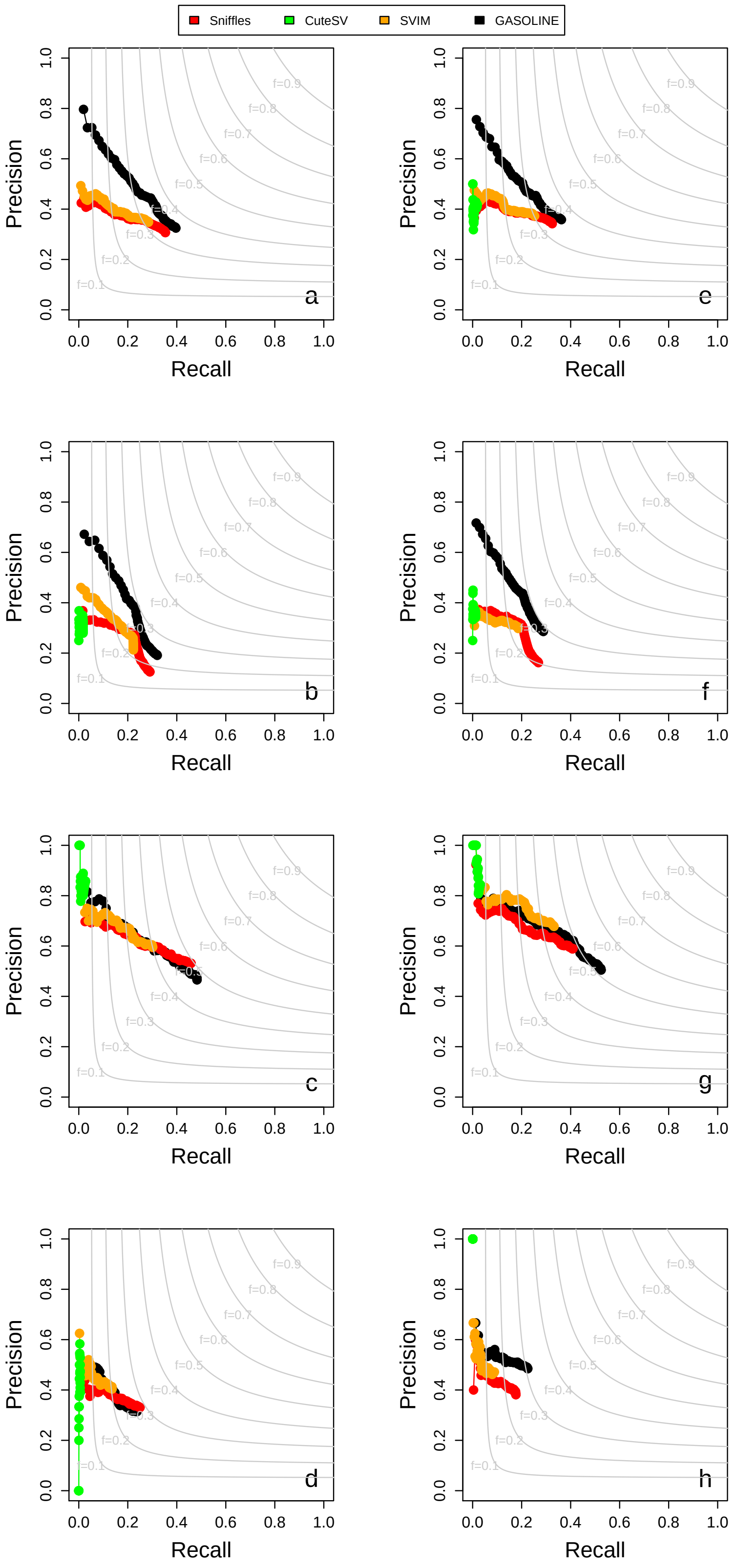

Supplementary Figure S12: Precision-Recall curves of GASOLINE and the other three tools as a function of number of supporting reads for NA24385 PacBio data downsampled at 5x. Panels (a-d) show the results for minimap2 alignment and (e-h) for NGMLR. Panels (a, e) for small deletions (< 500 bp), (b, f) for small insertions (< 500 bp), (a, e) for large deletions (> 500 bp), (a, e) for large insertions (> 500 bp). The curves in panels were obtained by ordering all the SVs as a function of number of supporting reads and calculating precision and recall including SVs with decreasing number of reads.

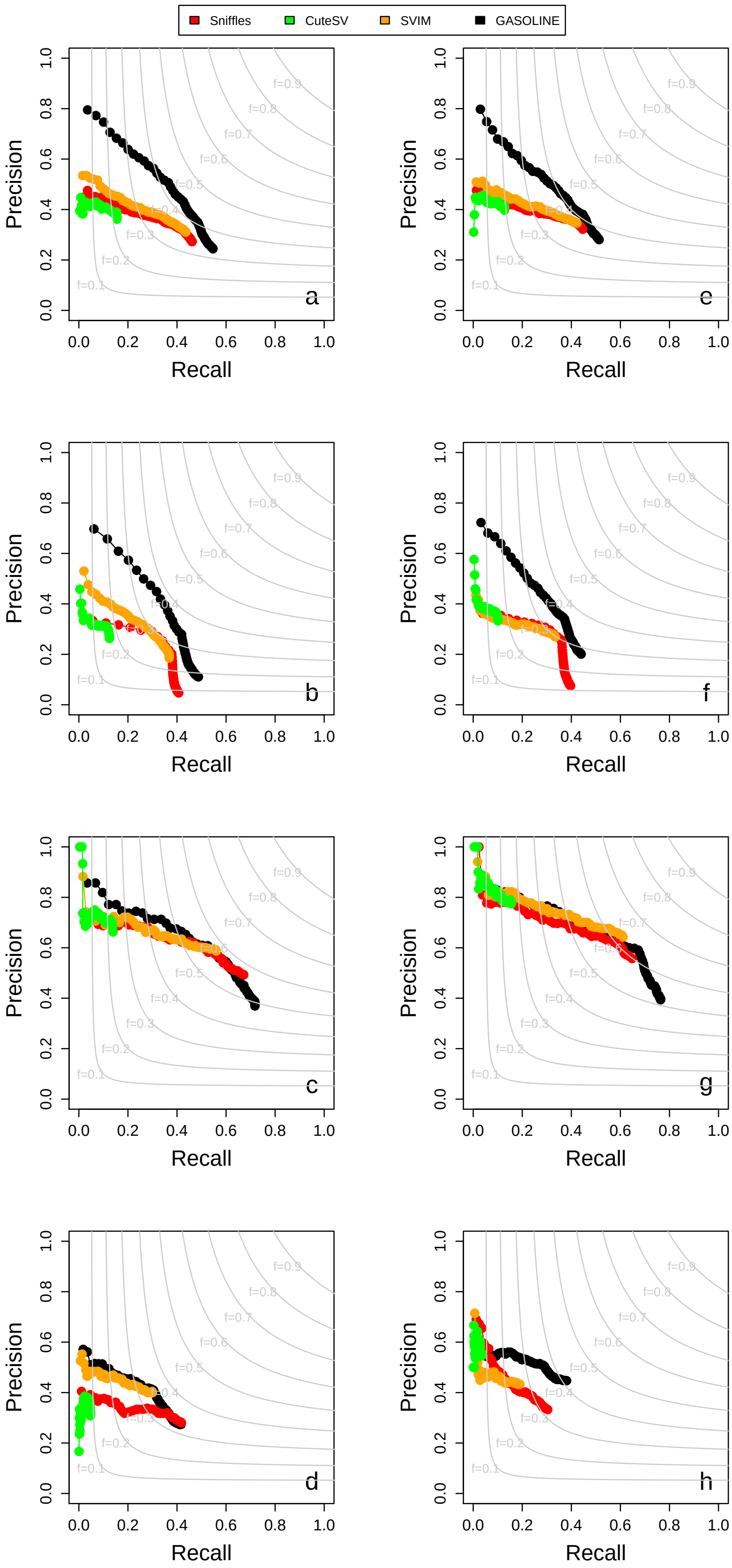

Supplementary Figure S13: Precision-Recall curves of GASOLINE and the other three tools as a function of number of supporting reads for NA24385 PacBio data downsampled at 10x. Panels (a-d) show the results for minimap2 alignment and (e-h) for NGMLR. Panels (a, e) for small deletions (< 500 bp), (b, f) for small insertions (< 500 bp), (a, e) for large deletions (> 500 bp), (a, e) for large insertions (> 500 bp). The curves in panels were obtained by ordering all the SVs as a function of number of supporting reads and calculating precision and recall including SVs with decreasing number of reads.

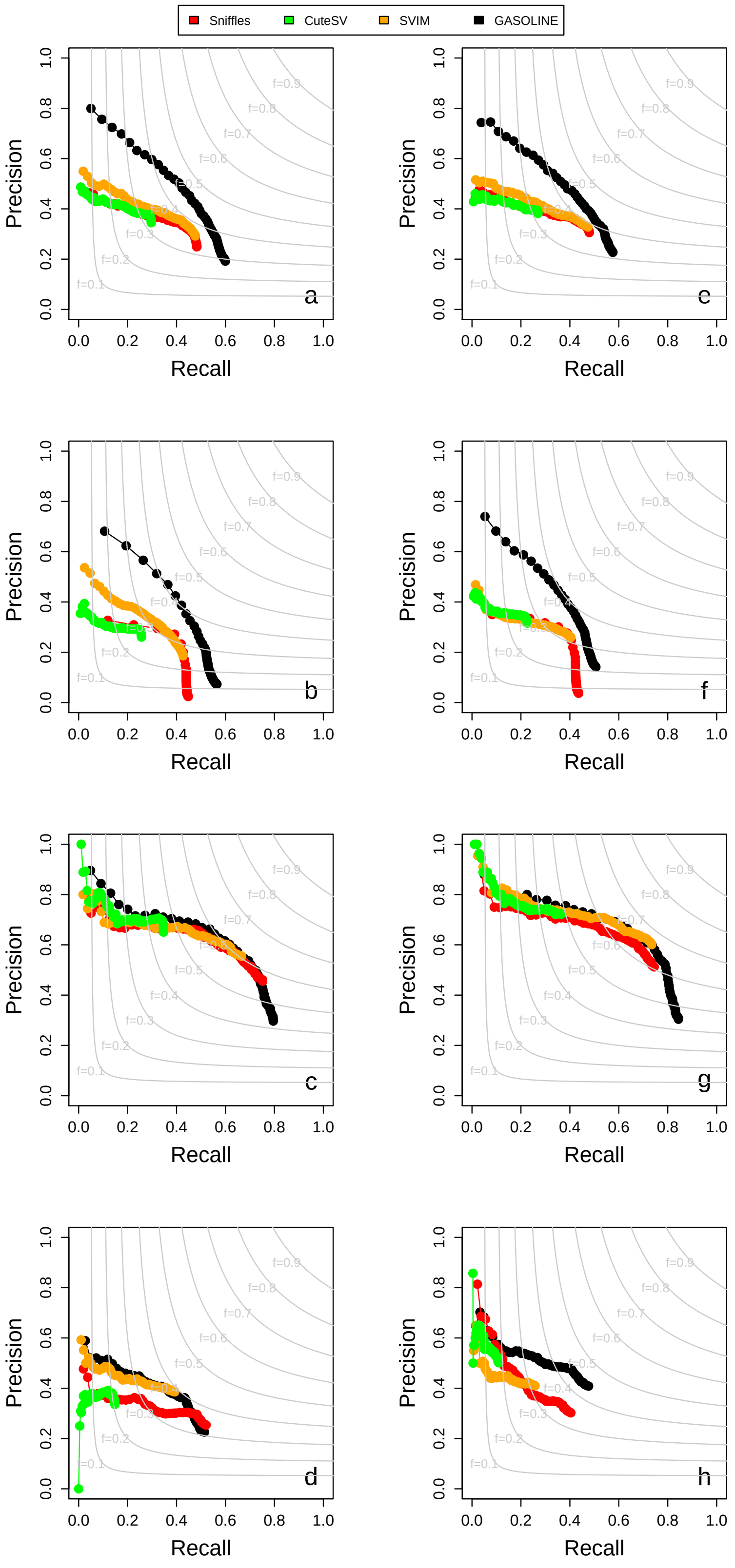

Supplementary Figure S14: Precision-Recall curves of GASOLINE and the other three tools as a function of number of supporting reads for NA24385 PacBio data downsampled at 15x. Panels (a-d) show the results for minimap2 alignment and (e-h) for NGMLR. Panels (a, e) for small deletions (< 500 bp), (b, f) for small insertions (< 500 bp), (a, e) for large deletions (> 500 bp), (a, e) for large insertions (> 500 bp). The curves in panels were obtained by ordering all the SVs as a function of number of supporting reads and calculating precision and recall including SVs with decreasing number of reads.

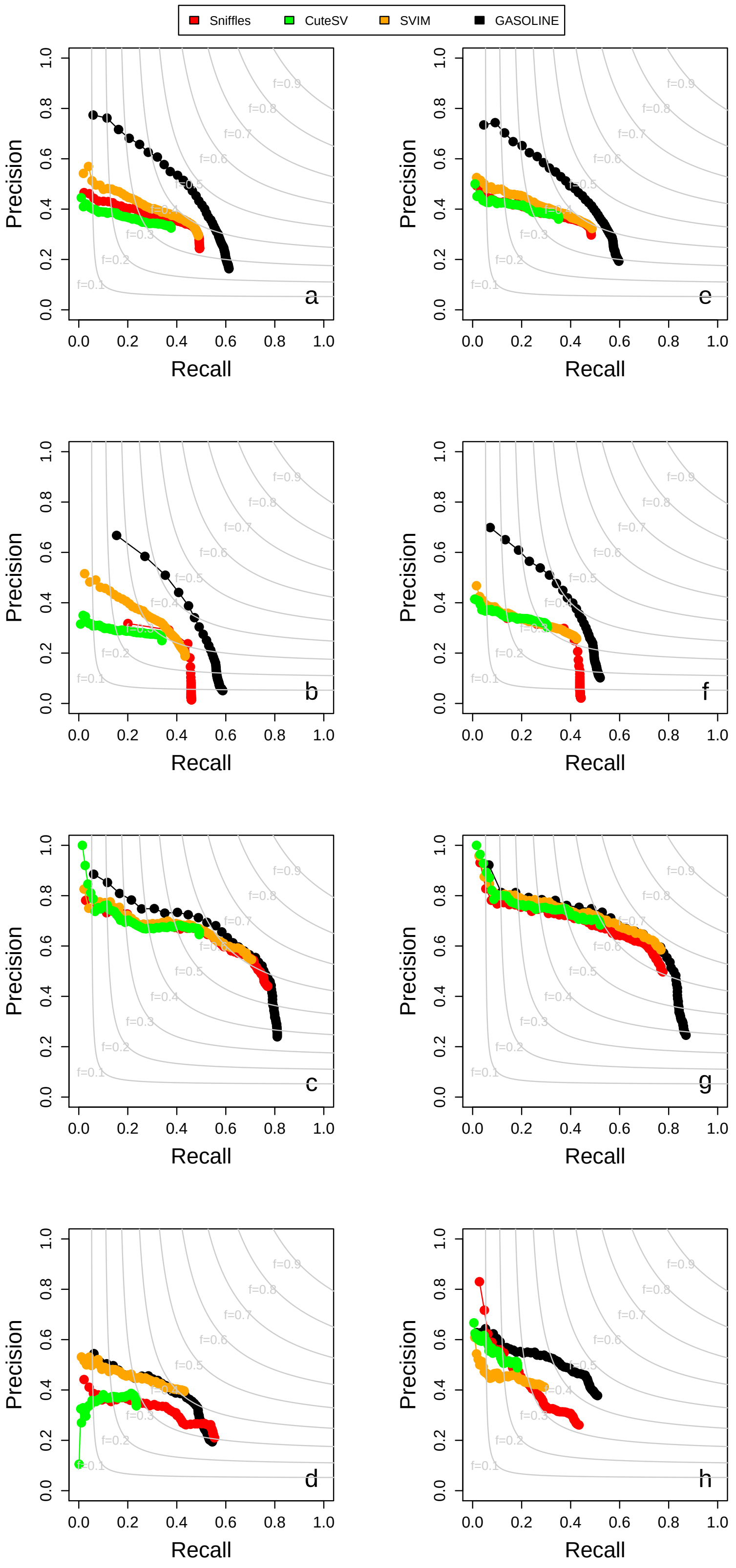

Supplementary Figure S15: Precision-Recall curves of GASOLINE and the other three tools as a function of number of supporting reads for NA24385 PacBio data downsampled at 20x. Panels (a-d) show the results for minimap2 alignment and (e-h) for NGMLR. Panels (a, e) for small deletions (< 500 bp), (b, f) for small insertions (< 500 bp), (a, e) for large deletions (> 500 bp), (a, e) for large insertions (> 500 bp). The curves in panels were obtained by ordering all the SVs as a function of number of supporting reads and calculating precision and recall including SVs with decreasing number of reads.

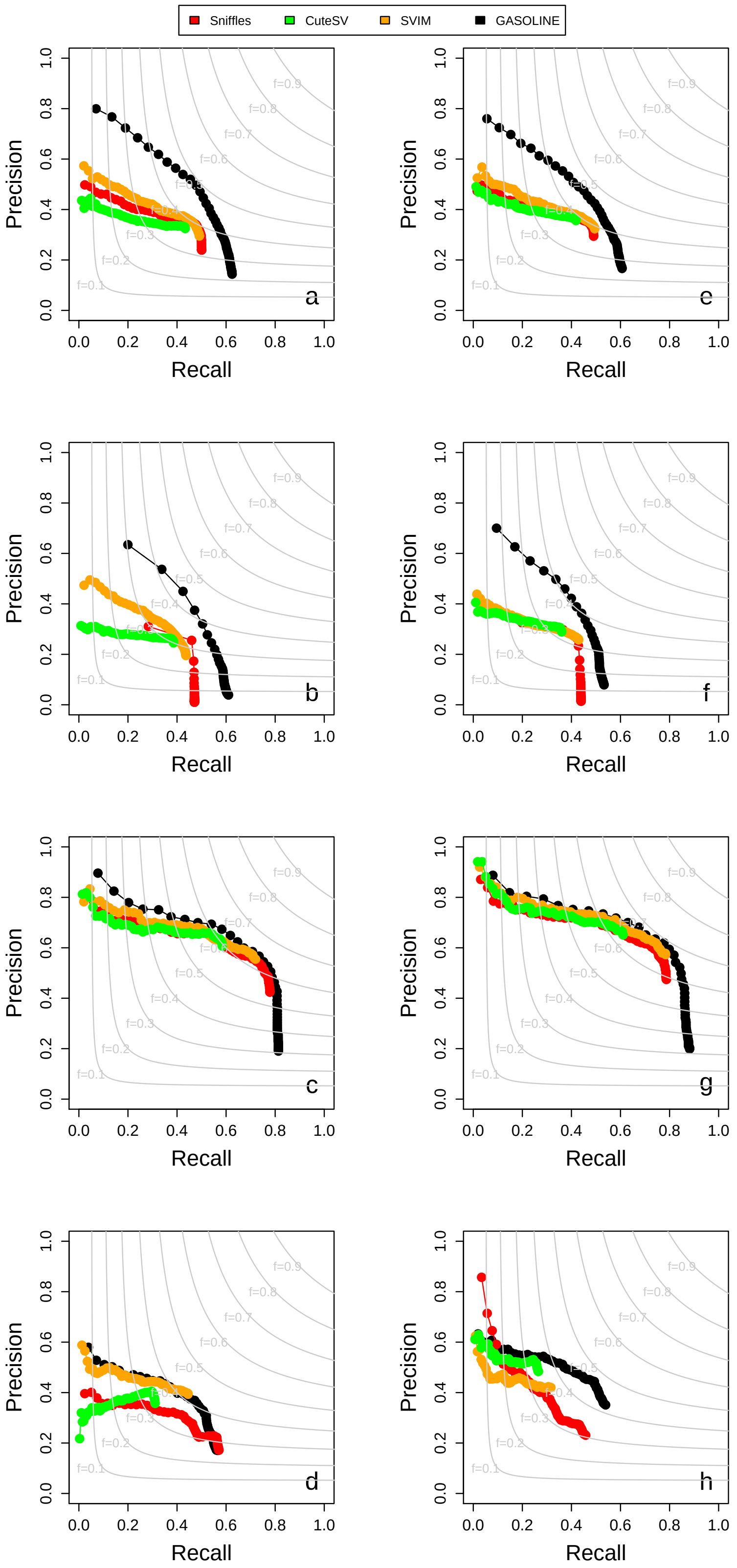

Supplementary Figure S16: Precision-Recall curves of GASOLINE and the other three tools as a function of number of supporting reads for NA24385 PacBio data downsampled at 25x. Panels (a-d) show the results for minimap2 alignment and (e-h) for NGMLR. Panels (a, e) for small deletions (< 500 bp), (b, f) for small insertions (< 500 bp), (a, e) for large deletions (> 500 bp), (a, e) for large insertions (> 500 bp). The curves in panels were obtained by ordering all the SVs as a function of number of supporting reads and calculating precision and recall including SVs with decreasing number of reads.

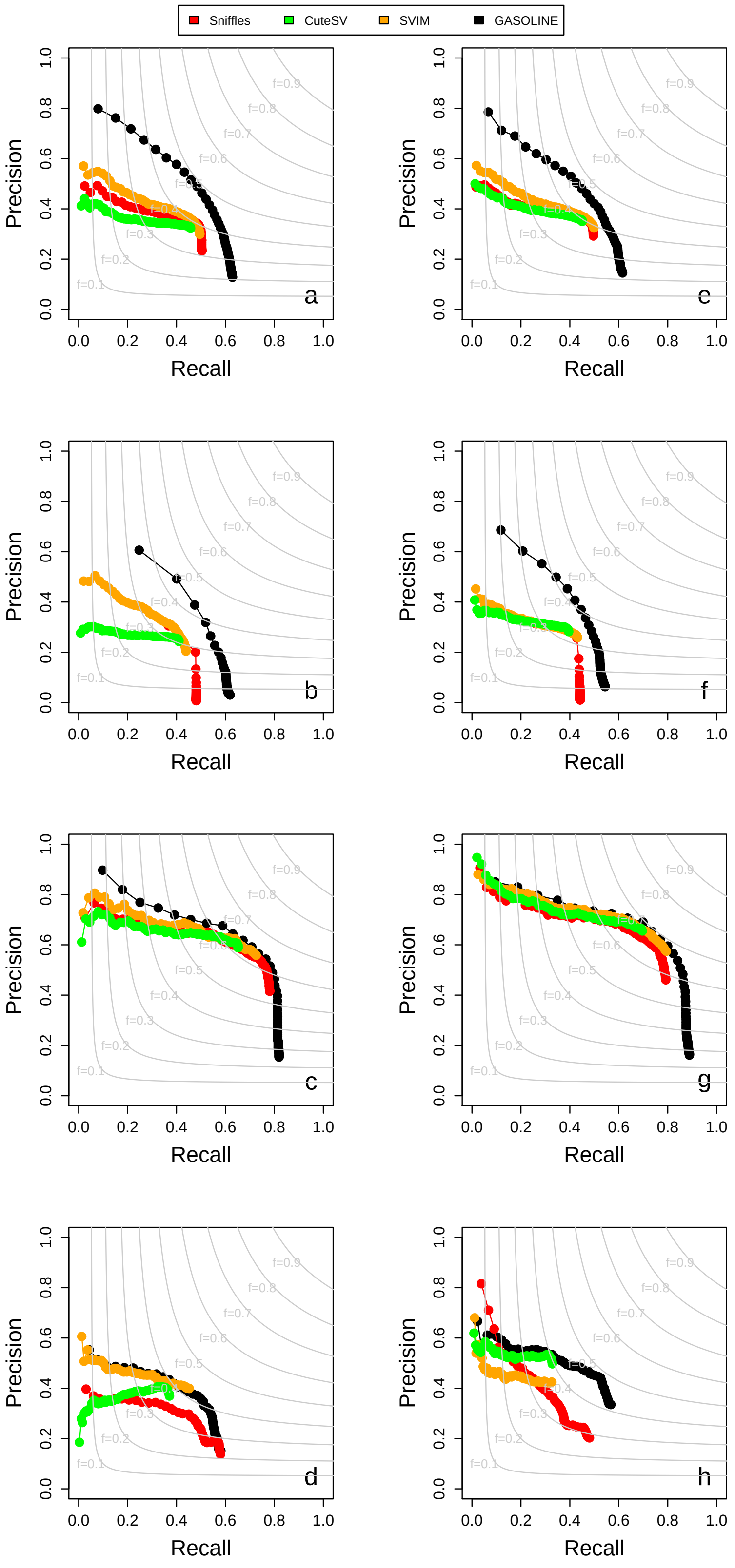

Supplementary Figure S17: Precision-Recall curves of GASOLINE and the other three tools as a function of number of supporting reads for NA24385 PacBio data downsampled at 30x. Panels (a-d) show the results for minimap2 alignment and (e-h) for NGMLR. Panels (a, e) for small deletions (< 500 bp), (b, f) for small insertions (< 500 bp), (a, e) for large deletions (> 500 bp), (a, e) for large insertions (> 500 bp). The curves in panels were obtained by ordering all the SVs as a function of number of supporting reads and calculating precision and recall including SVs with decreasing number of reads.

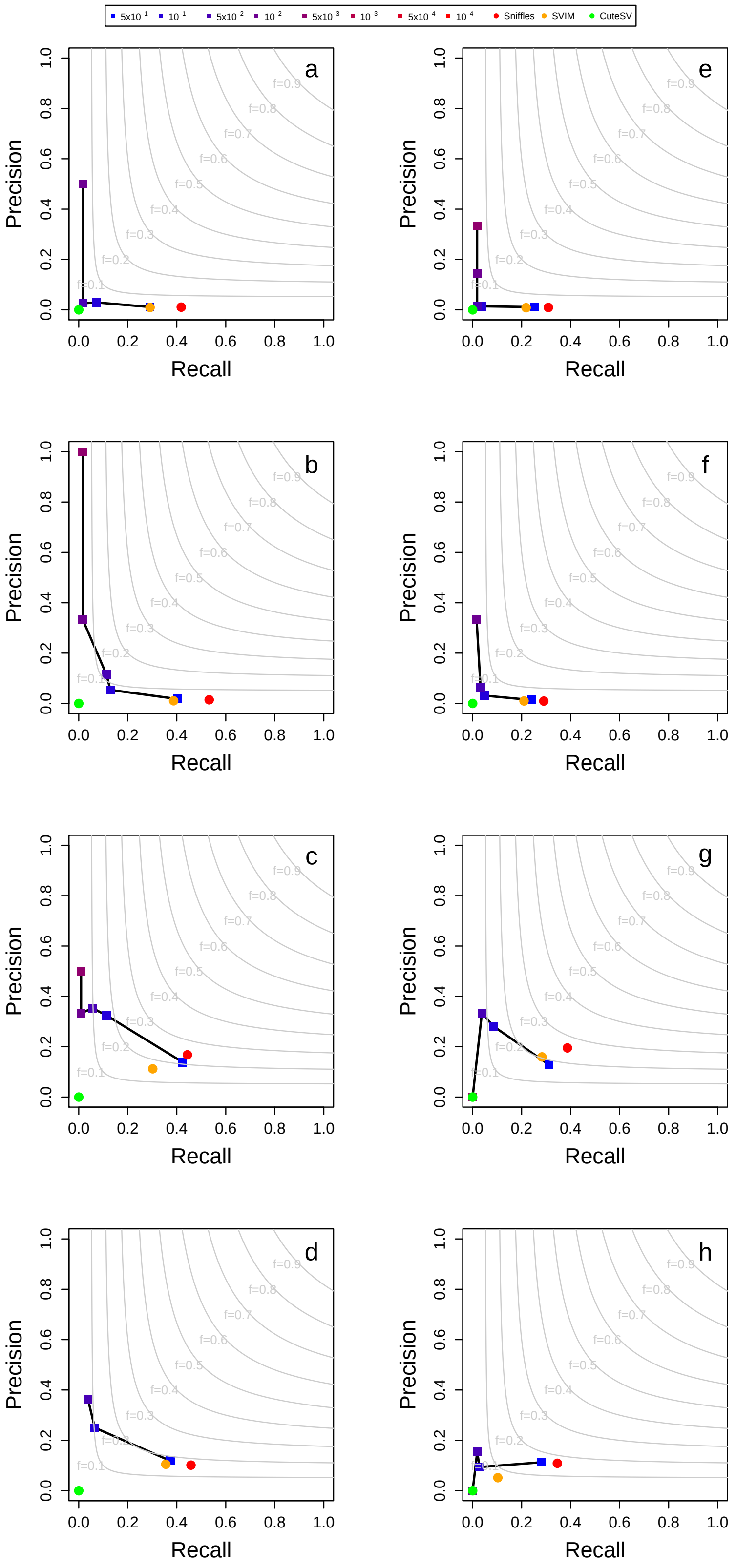

Supplementary Figure S18: Precision and recall obtained by GASOLINE and the other three tools in the detection simulated somatic SVs from NA24385 ONT data downsampled at 5x. Panels (a-d) show the results for minimap2 alignment and (e-h) for NGMLR. Panels (a, e) for small deletions (< 500 bp), (b, f) for small insertions (< 500 bp), (a, e) for large deletions (> 500 bp), (a, e) for large insertions (> 500 bp). The results for GASOLINE were reported for different somatic p-value thresholds (5x10^{-1}, 1x10^{-1}, 5x10^{-2}, 1x10^{-2}, 5x10^{-3}, 1x10^{-3}, 5x10^{-4}, 1x10^{-4}).

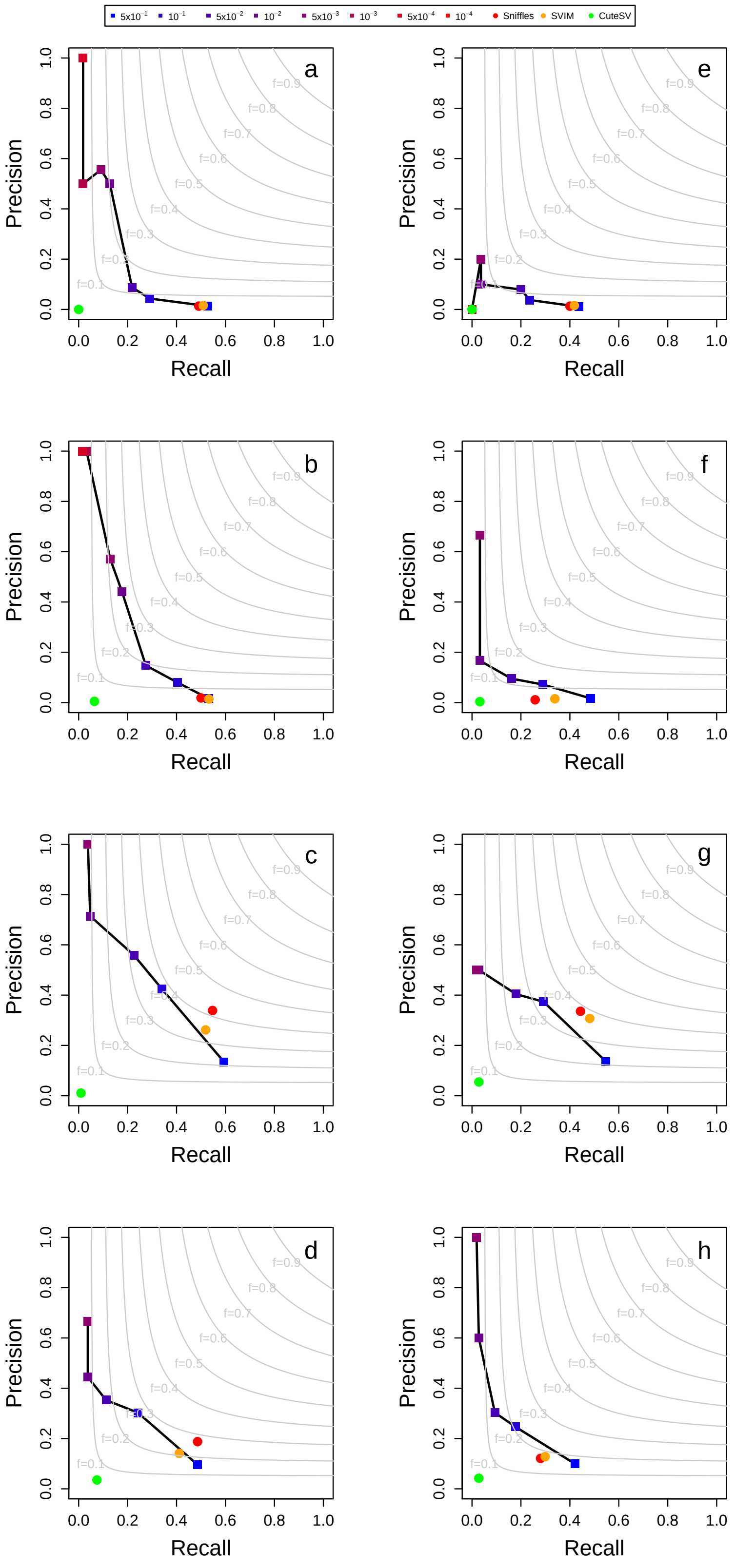

Supplementary Figure S19: Precision and recall obtained by GASOLINE and the other three tools in the detection simulated somatic SVs from NA24385 ONT data downsampled at 10x. Panels (a-d) show the results for minimap2 alignment and (e-h) for NGMLR. Panels (a, e) for small deletions (< 500 bp), (b, f) for small insertions (< 500 bp), (a, e) for large deletions (> 500 bp), (a, e) for large insertions (> 500 bp). The results for GASOLINE were reported for different somatic p-value thresholds ($5x10^{-1}$, $1x10^{-1}$, $5x10^{-2}$, $1x10^{-2}$, $5x10^{-3}$, $1x10^{-3}$, $5x10^{-4}$, $1x10^{-4}$).

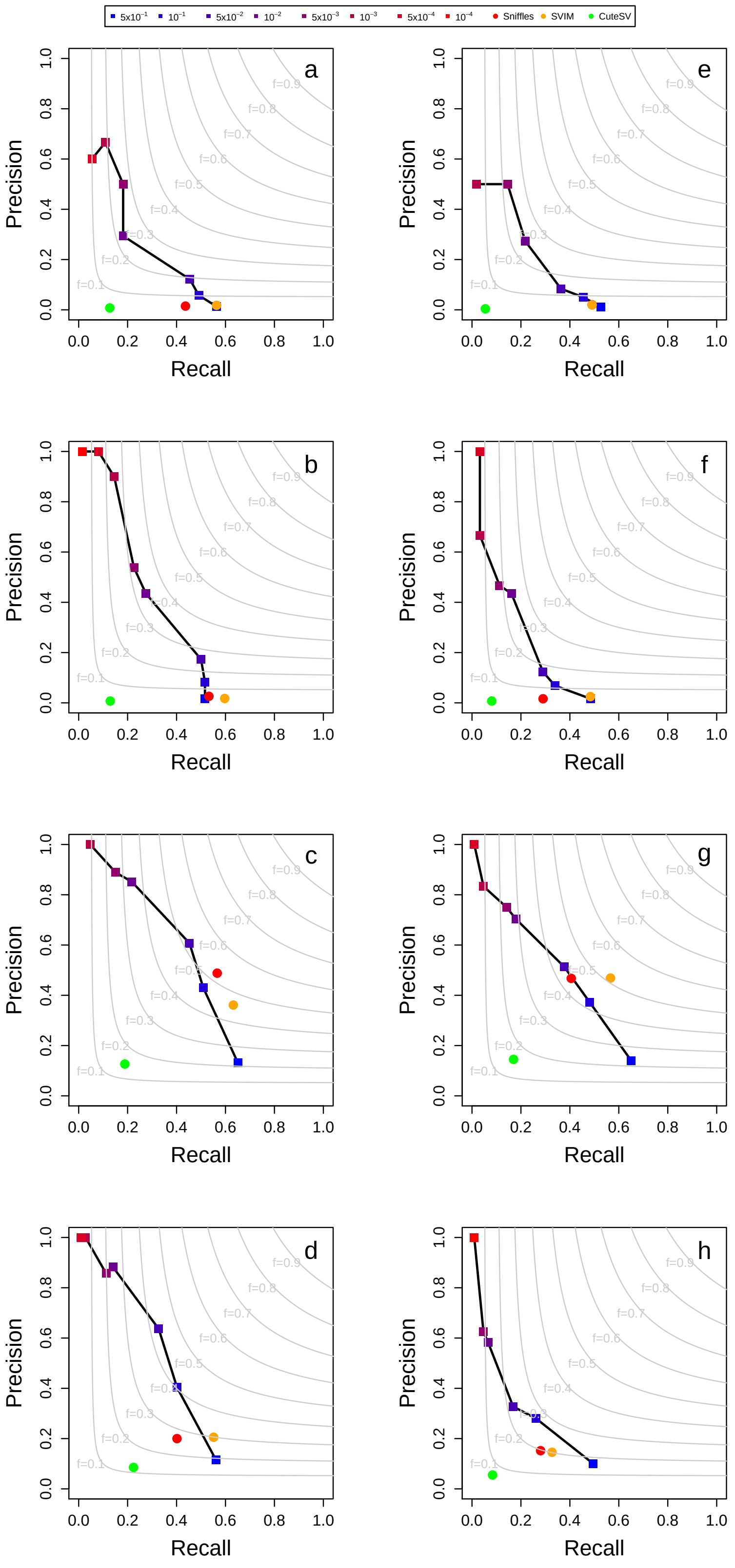

Supplementary Figure S20: Precision and recall obtained by GASOLINE and the other three tools in the detection simulated somatic SVs from NA24385 ONT data downsampled at 15x. Panels (a-d) show the results for minimap2 alignment and (e-h) for NGMLR. Panels (a, e) for small deletions (< 500 bp), (b, f) for small insertions (< 500 bp), (a, e) for large deletions (> 500 bp), (a, e) for large insertions (> 500 bp). The results for GASOLINE were reported for different somatic p-value thresholds ($5x10^{-1}$, $1x10^{-1}$, $5x10^{-2}$, $1x10^{-2}$, $5x10^{-3}$, $1x10^{-3}$, $5x10^{-4}$, $1x10^{-4}$).

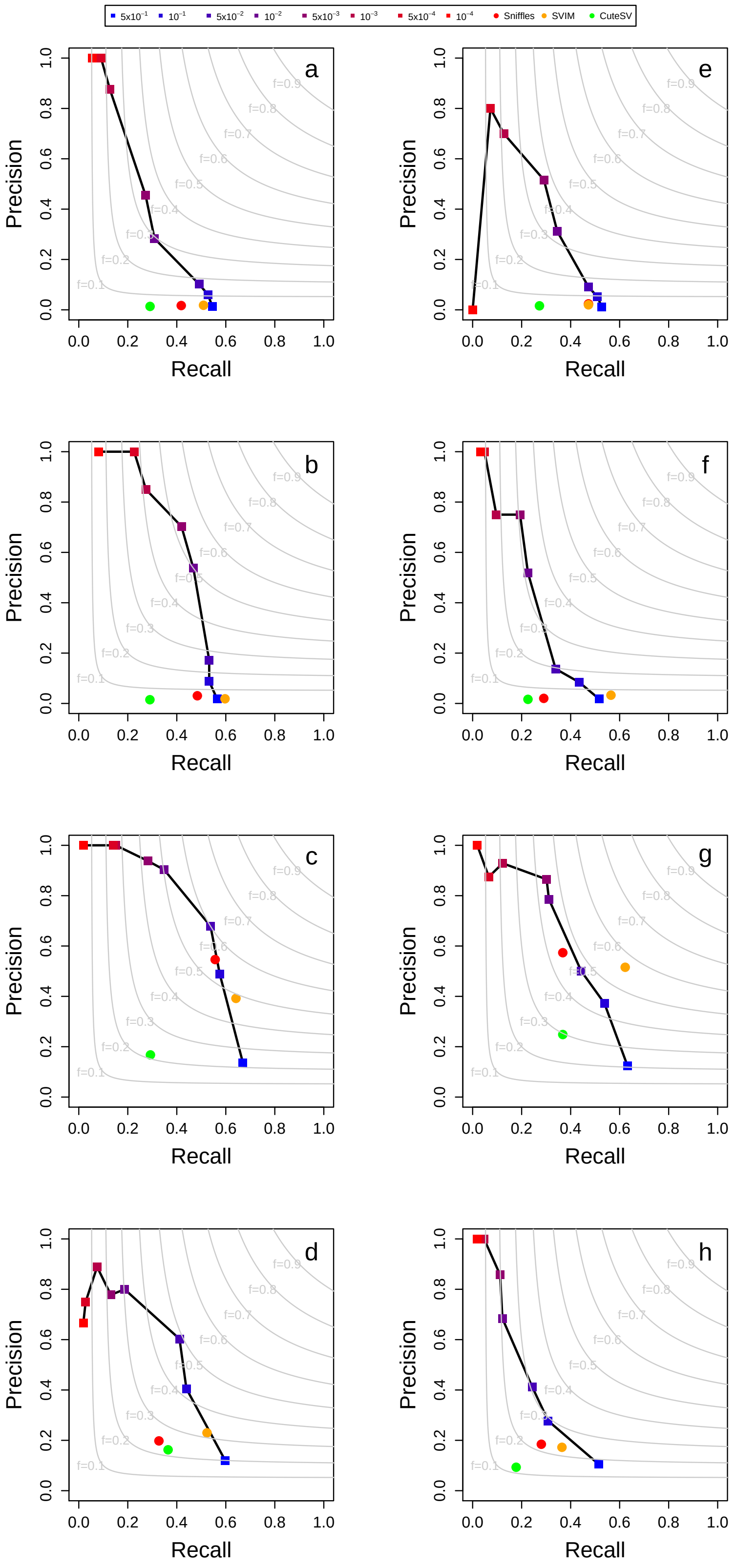

Supplementary Figure S21: Precision and recall obtained by GASOLINE and the other three tools in the detection simulated somatic SVs from NA24385 ONT data downsampled at 20x. Panels (a-d) show the results for minimap2 alignment and (e-h) for NGMLR. Panels (a, e) for small deletions (< 500 bp), (b, f) for small insertions (< 500 bp), (a, e) for large deletions (> 500 bp), (a, e) for large insertions (> 500 bp). The results for GASOLINE were reported for different somatic p-value thresholds ($5x10^{-1}$, $1x10^{-1}$, $5x10^{-2}$, $1x10^{-2}$, $5x10^{-3}$, $1x10^{-3}$, $5x10^{-4}$, $1x10^{-4}$).

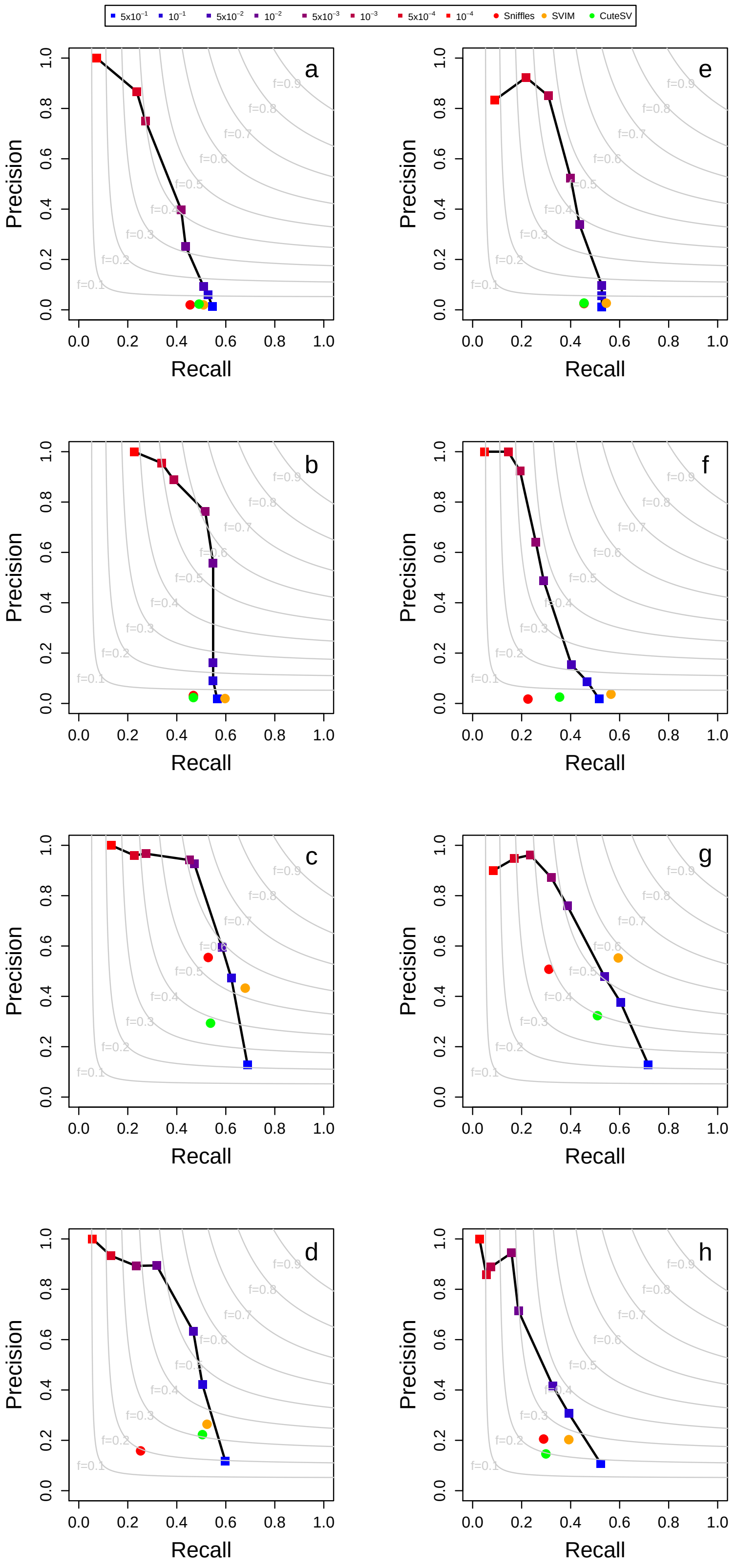

Supplementary Figure S22: Precision and recall obtained by GASOLINE and the other three tools in the detection simulated somatic SVs from NA24385 ONT data downsampled at 25x. Panels (a-d) show the results for minimap2 alignment and (e-h) for NGMLR. Panels (a, e) for small deletions (< 500 bp), (b, f) for small insertions (< 500 bp), (a, e) for large deletions (> 500 bp), (a, e) for large insertions (> 500 bp). The results for GASOLINE were reported for different somatic p-value thresholds (5x10^{-1}, 1x10^{-1}, 5x10^{-2}, 1x10^{-2}, 5x10^{-3}, 1x10^{-3}, 5x10^{-4}, 1x10^{-4}).

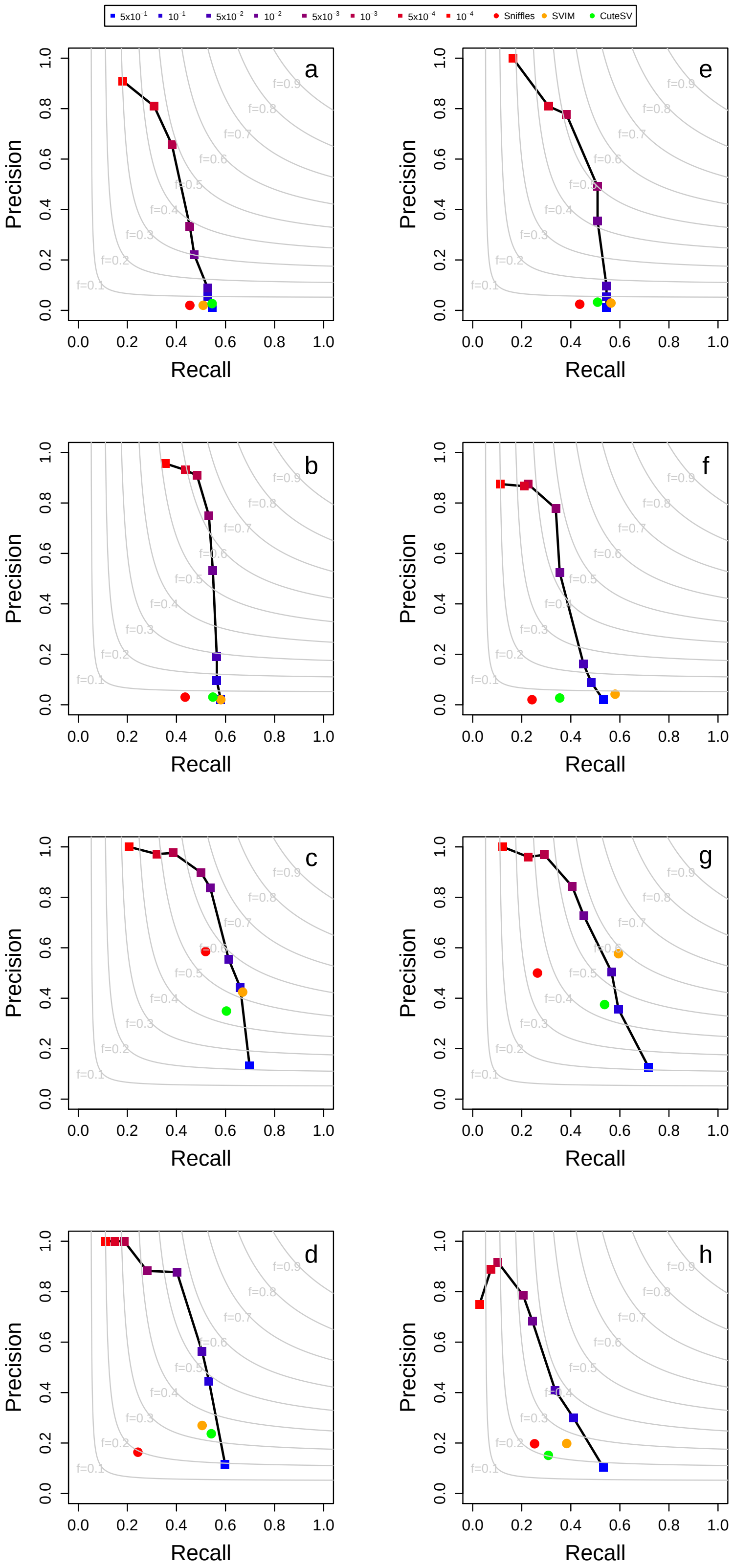

Supplementary Figure 23: Precision and recall obtained by GASOLINE and the other three tools in the detection simulated somatic SVs from NA24385 ONT data downsampled at 30x. Panels (a-d) show the results for minimap2 alignment and (e-h) for NGMLR. Panels (a, e) for small deletions (< 500 bp), (b, f) for small insertions (< 500 bp), (a, e) for large deletions (> 500 bp), (a, e) for large insertions (> 500 bp). The results for GASOLINE were reported for different somatic p-value thresholds ($5x10^{-1}$, $1x10^{-1}$, $5x10^{-2}$, $1x10^{-2}$, $5x10^{-3}$, $1x10^{-3}$, $5x10^{-4}$, $1x10^{-4}$).

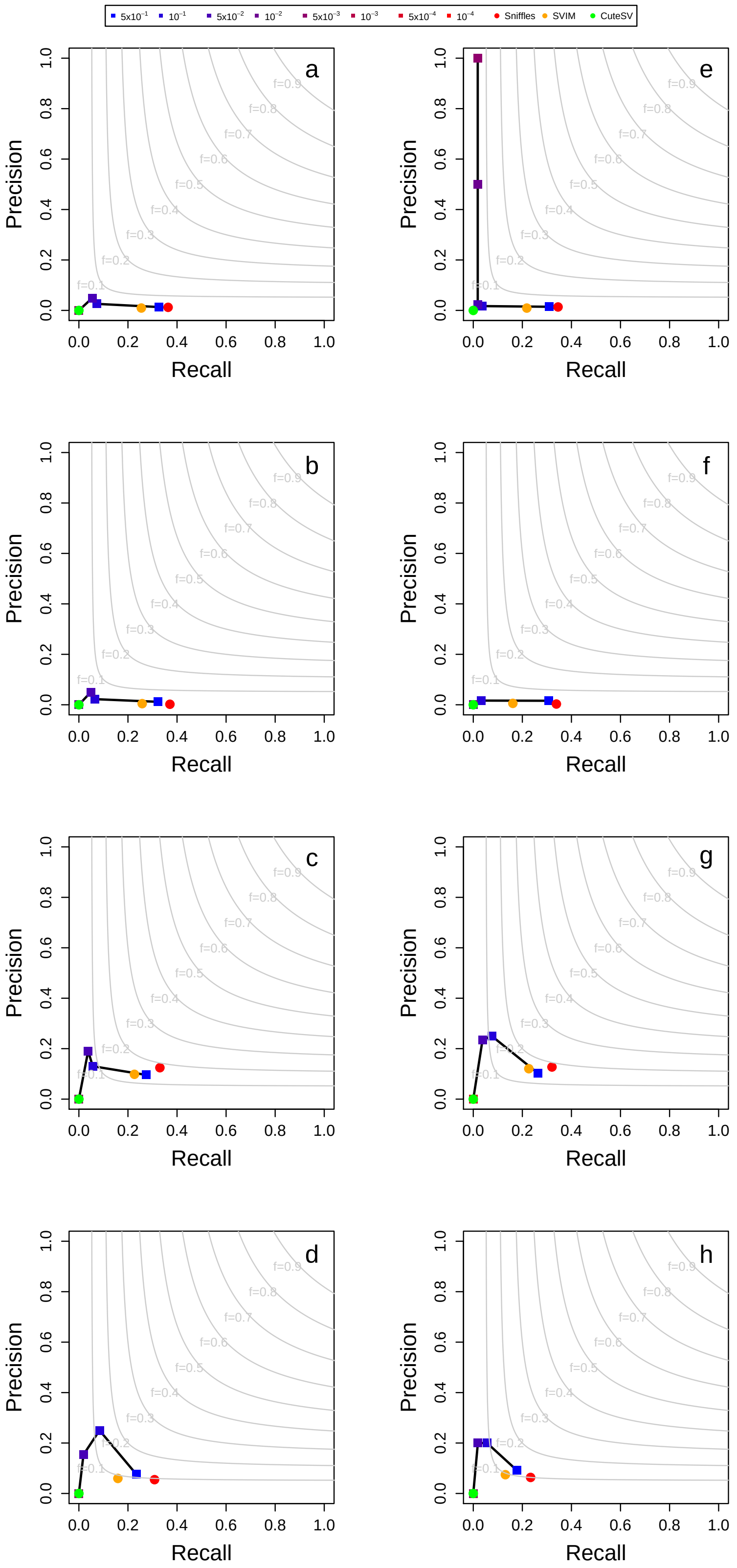

Supplementary Figure S24: Precision and recall obtained by GASOLINE and the other three tools in the detection simulated somatic SVs from NA24385 PacBio data downsampled at 5x. Panels (a-d) show the results for minimap2 alignment and (e-h) for NGMLR. Panels (a, e) for small deletions (< 500 bp), (b, f) for small insertions (< 500 bp), (a, e) for large deletions (> 500 bp), (a, e) for large insertions (> 500 bp). The results for GASOLINE were reported for different somatic p-value thresholds (5x10^{-1}, 1x10^{-1}, 5x10^{-2}, 1x10^{-2}, 5x10^{-3}, 1x10^{-3}, 5x10^{-4}, 1x10^{-4}).

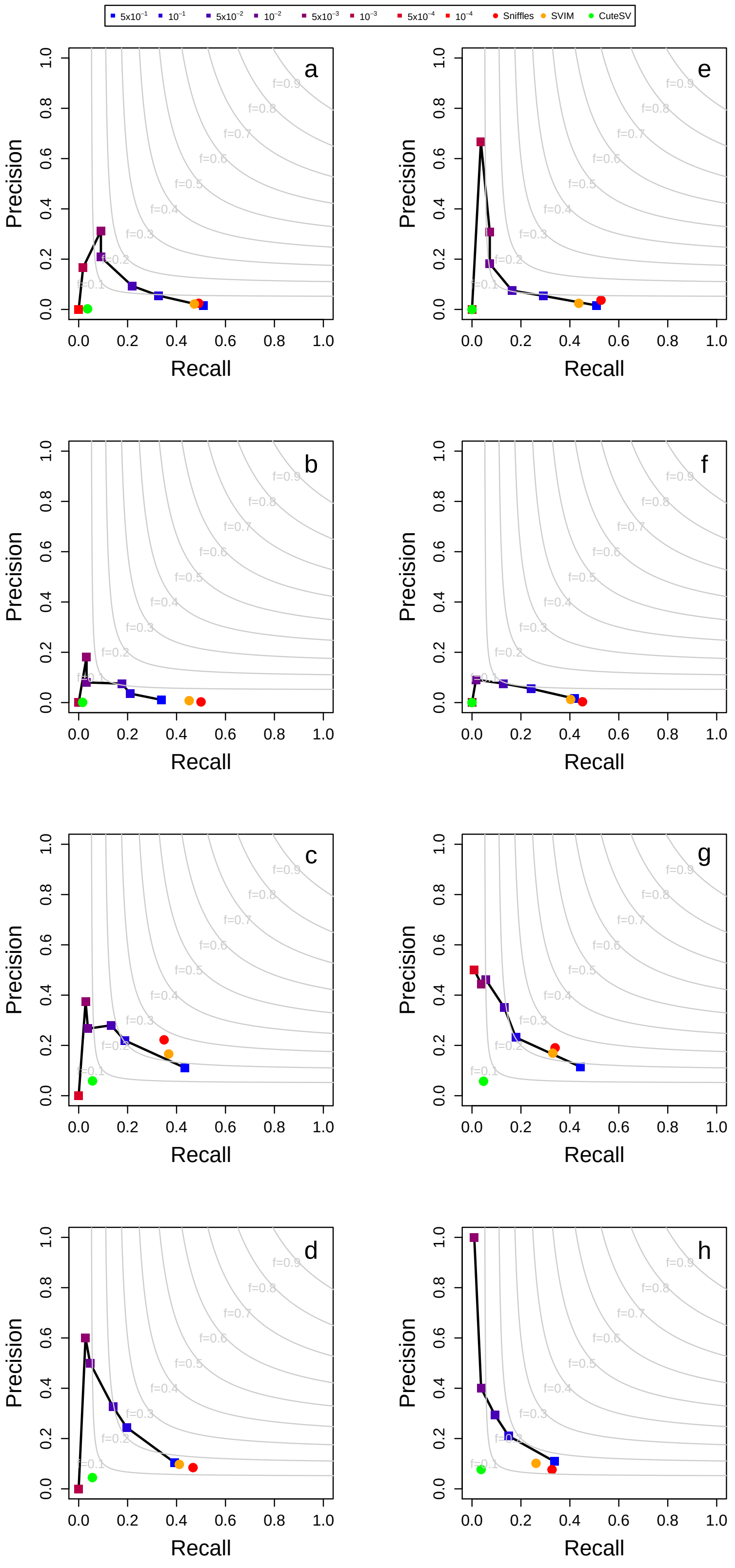

Supplementary Figure S25: Precision and recall obtained by GASOLINE and the other three tools in the detection simulated somatic SVs from NA24385 PacBio data downsampled at 10x. Panels (a-d) show the results for minimap2 alignment and (e-h) for NGMLR. Panels (a, e) for small deletions (< 500 bp), (b, f) for small insertions (< 500 bp), (a, e) for large deletions (> 500 bp), (a, e) for large insertions (> 500 bp). The results for GASOLINE were reported for different somatic p-value thresholds (5x10^{-1}, 1x10^{-1}, 5x10^{-2}, 1x10^{-2}, 5x10^{-3}, 1x10^{-3}, 5x10^{-4}, 1x10^{-4}).

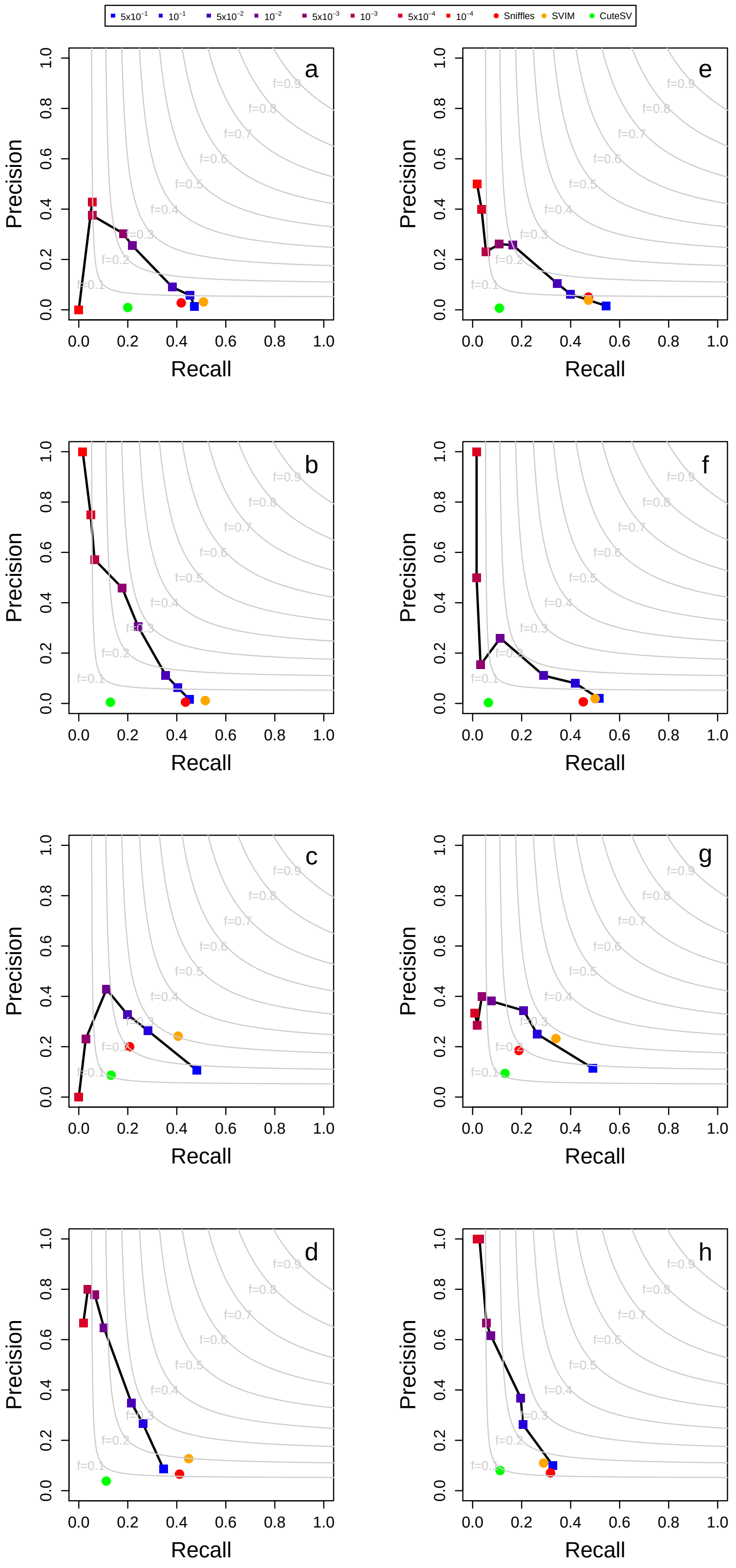

Supplementary Figure S26: Precision and recall obtained by GASOLINE and the other three tools in the detection simulated somatic SVs from NA24385 PacBio data downsampled at 15x. Panels (a-d) show the results for minimap2 alignment and (e-h) for NGMLR. Panels (a, e) for small deletions (< 500 bp), (b, f) for small insertions (< 500 bp), (a, e) for large deletions (> 500 bp), (a, e) for large insertions (> 500 bp). The results for GASOLINE were reported for different somatic p-value thresholds (5x10^{-1}, 1x10^{-1}, 5x10^{-2}, 1x10^{-2}, 5x10^{-3}, 1x10^{-3}, 5x10^{-4}, 1x10^{-4}).

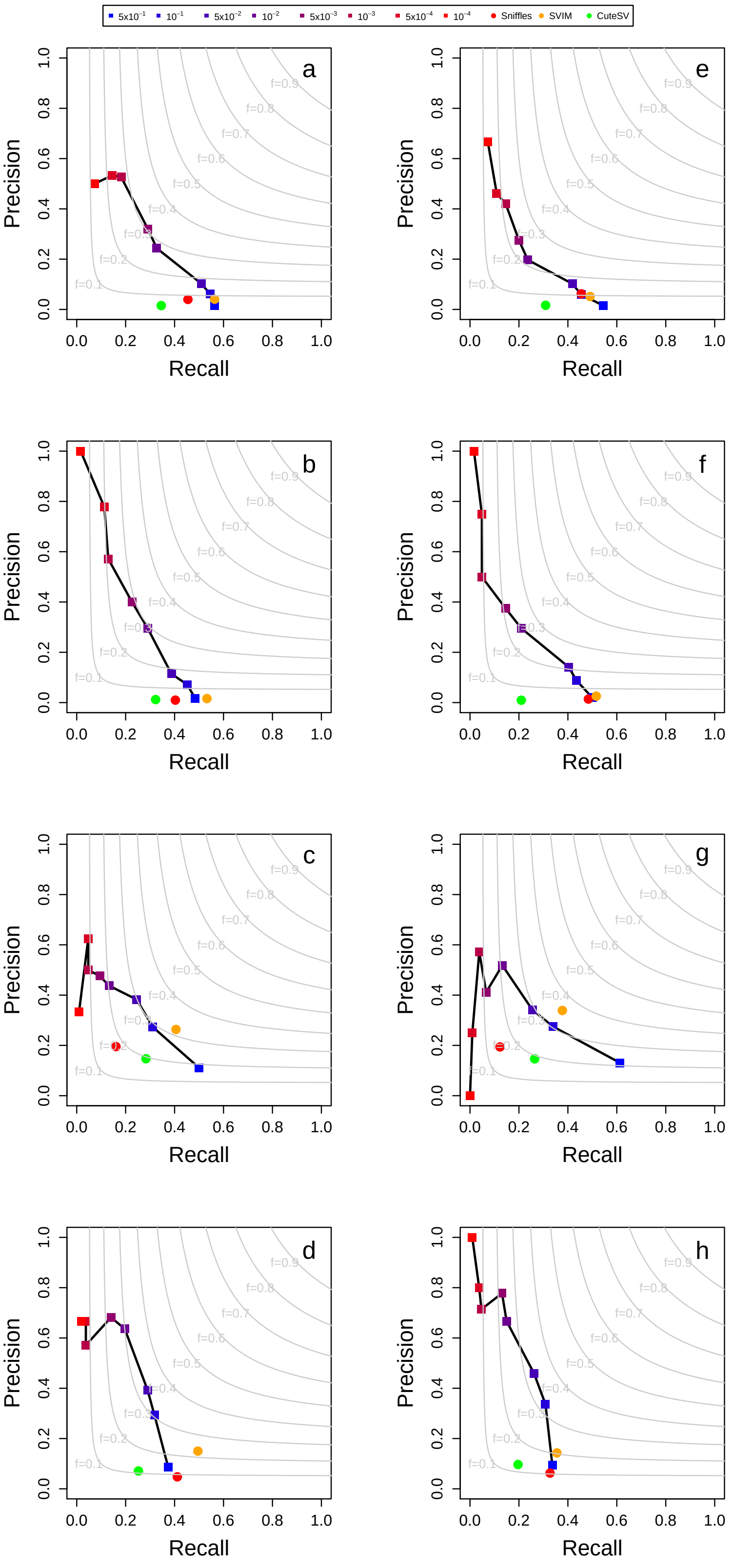

Supplementary Figure S27: Precision and recall obtained by GASOLINE and the other three tools in the detection simulated somatic SVs from NA24385 PacBio data downsampled at 20x. Panels (a-d) show the results for minimap2 alignment and (e-h) for NGMLR. Panels (a, e) for small deletions (< 500 bp), (b, f) for small insertions (< 500 bp), (a, e) for large deletions (> 500 bp), (a, e) for large insertions (> 500 bp). The results for GASOLINE were reported for different somatic p-value thresholds ($5x10^{-1}$, $1x10^{-1}$, $5x10^{-2}$, $1x10^{-2}$, $5x10^{-3}$, $1x10^{-3}$, $5x10^{-4}$, $1x10^{-4}$).

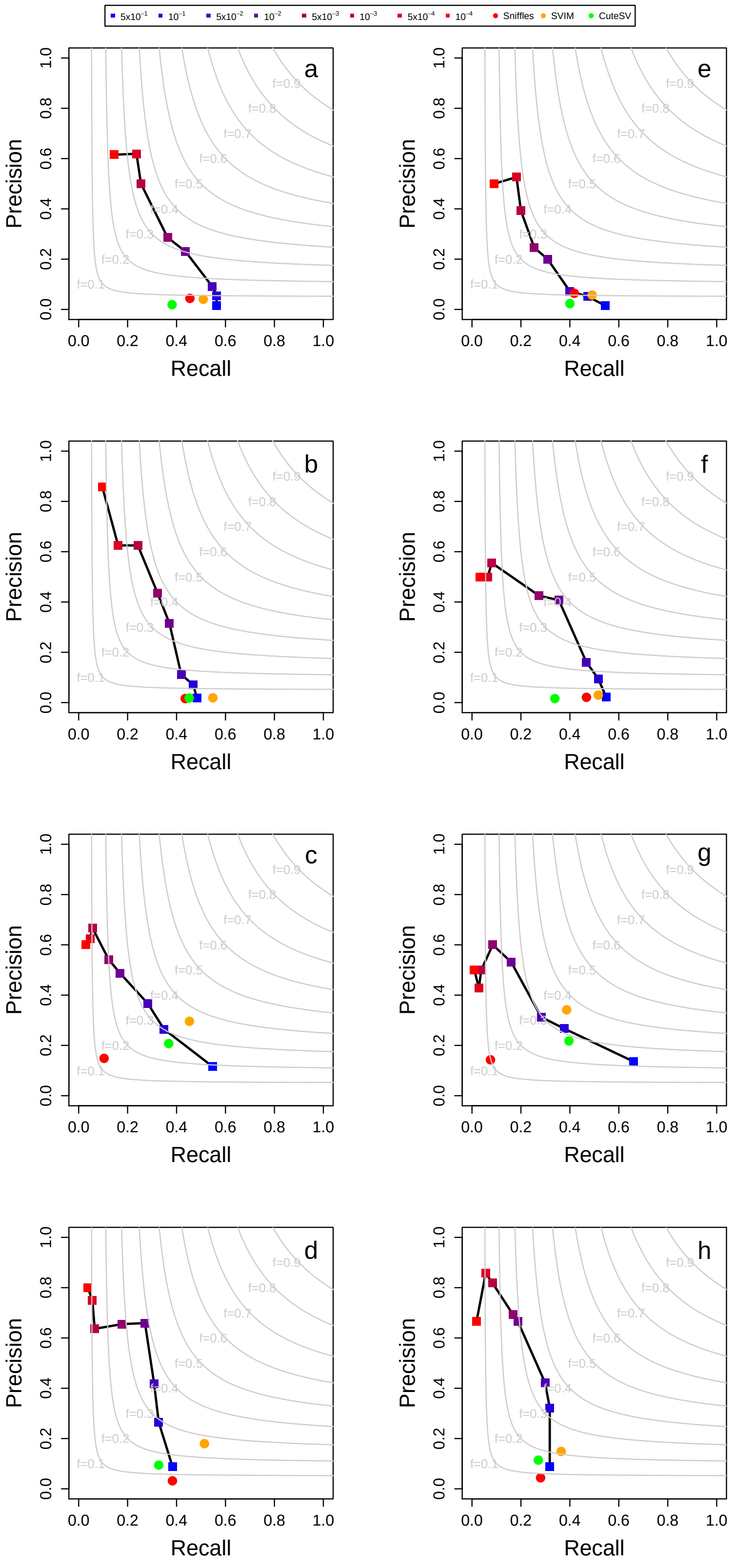

Supplementary Figure S28: Precision and recall obtained by GASOLINE and the other three tools in the detection simulated somatic SVs from NA24385 PacBio data downsampled at 25x. Panels (a-d) show the results for minimap2 alignment and (e-h) for NGMLR. Panels (a, e) for small deletions (< 500 bp), (b, f) for small insertions (< 500 bp), (a, e) for large deletions (> 500 bp), (a, e) for large insertions (> 500 bp). The results for GASOLINE were reported for different somatic p-value thresholds ($5x10^{-1}$, $1x10^{-1}$, $5x10^{-2}$, $1x10^{-2}$, $5x10^{-3}$, $1x10^{-3}$, $5x10^{-4}$, $1x10^{-4}$).

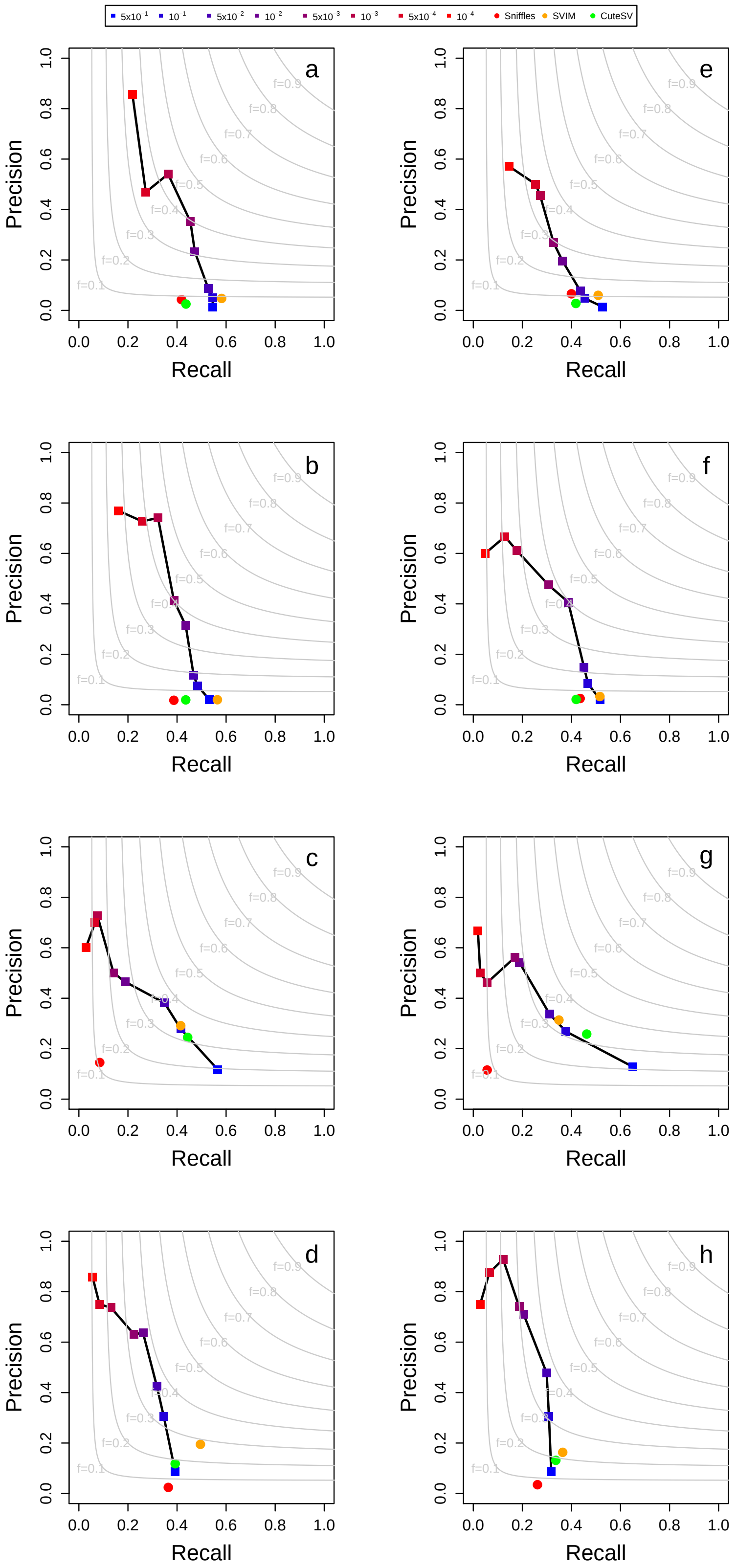

Supplementary Figure S29: Precision and recall obtained by GASOLINE and the other three tools in the detection simulated somatic SVs from NA24385 PacBio data downsampled at 30x. Panels (a-d) show the results for minimap2 alignment and (e-h) for NGMLR. Panels (a, e) for small deletions (< 500 bp), (b, f) for small insertions (< 500 bp), (a, e) for large deletions (> 500 bp), (a, e) for large insertions (> 500 bp). The results for GASOLINE were reported for different somatic p-value thresholds ($5x10^{-1}$, $1x10^{-1}$, $5x10^{-2}$, $1x10^{-2}$, $5x10^{-3}$, $1x10^{-3}$, $5x10^{-4}$, $1x10^{-4}$).

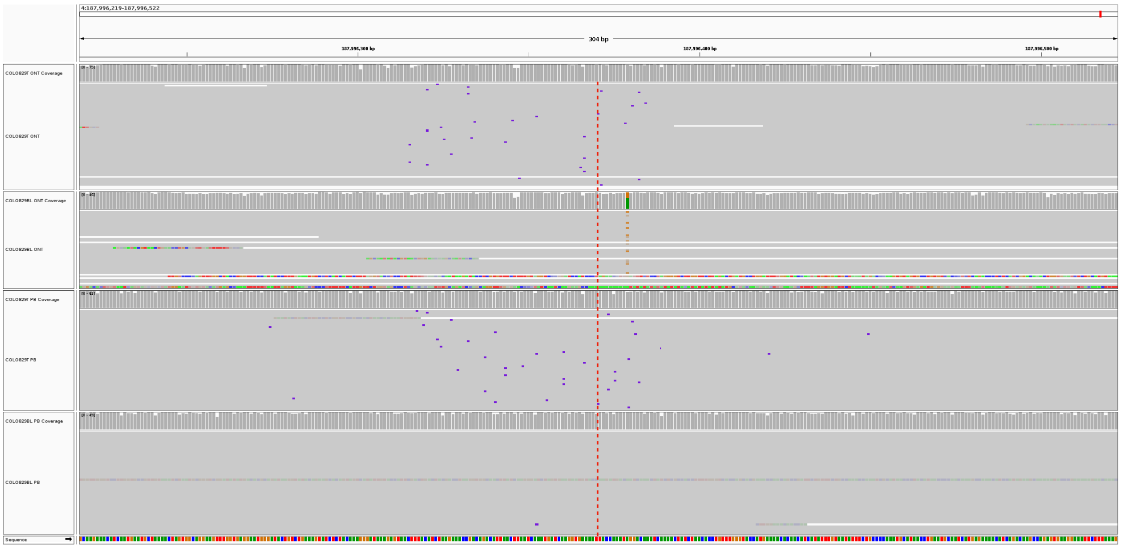

Supplementary Figure S30: IGV plot of the 52 bp insertion detected by GASOLINE on COLO829 sample at 4:187996371. Gray horizontal bars represent aligned reads. A black line represents a deletion, and a purple box is an insertion. Vertical dotted lines indicate the start (red) and end (green) positions predicted by GASOLINE. The plot confirms the presence of several insertion signatures in COLO829T and no signatures in COLO829BL for both ONT and PacBio data. The variant is considered true positive.

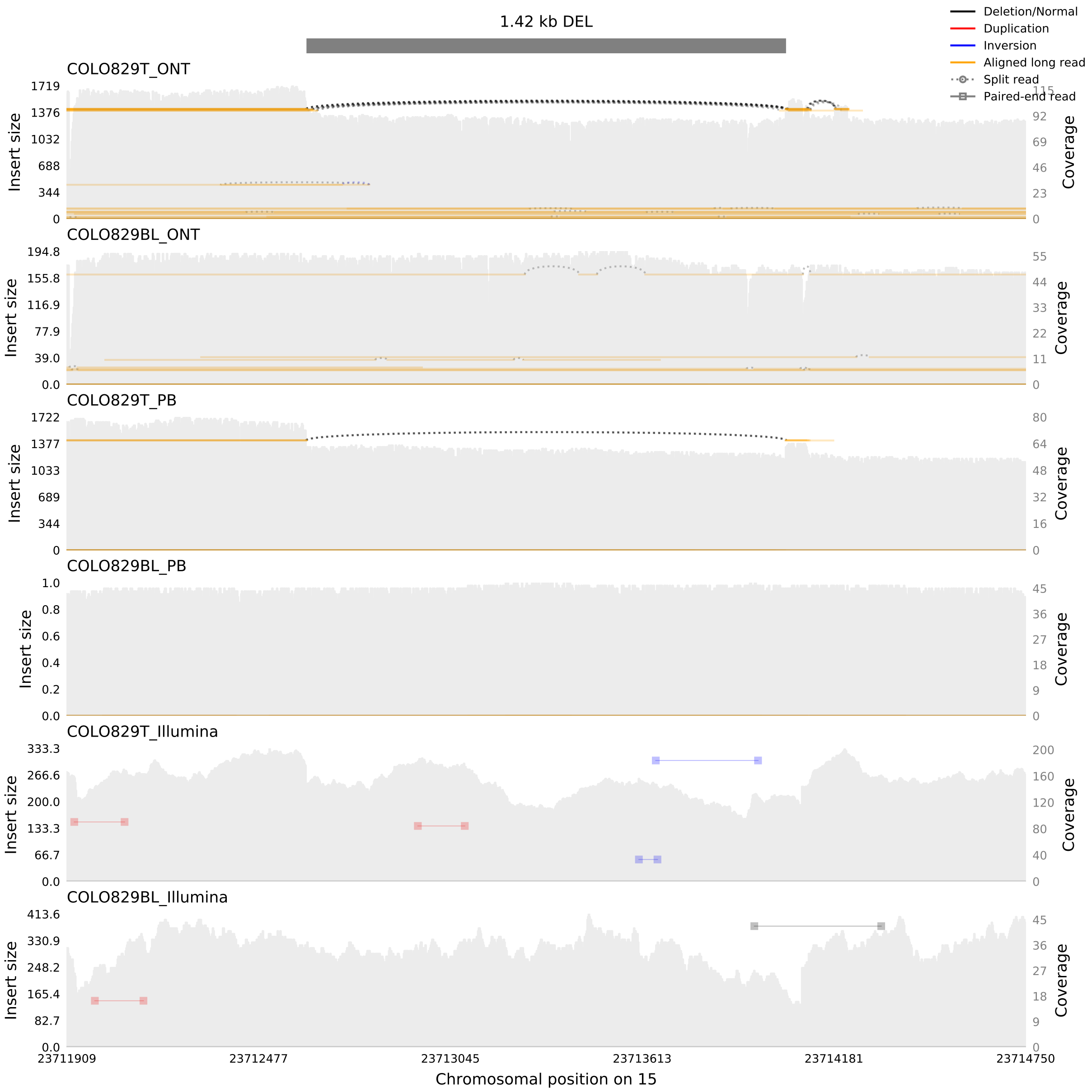

Supplementary Figure S31: Samplot of the 1.4 kb deletion detected by GASOLINE on COLO829 sample at 15:23712619-23714040. Reads are indicated by horizontal orange lines. Split alignments are indicated by color-coded dotted lines (black for deletions, red for duplications, blue for inversions) that allow to clearly and quickly distinguish between normal reads and reads supporting different SV types. The coverage for the region is shown with the gray-filled background, which is split into map quality above or below a user-defined threshold (in dark or light gray respectively). Split alignment signatures confirms the presence of the deletion in COLO829T and the absence in COLO829BL for both ONT and PacBio data. The variant is considered true positive.

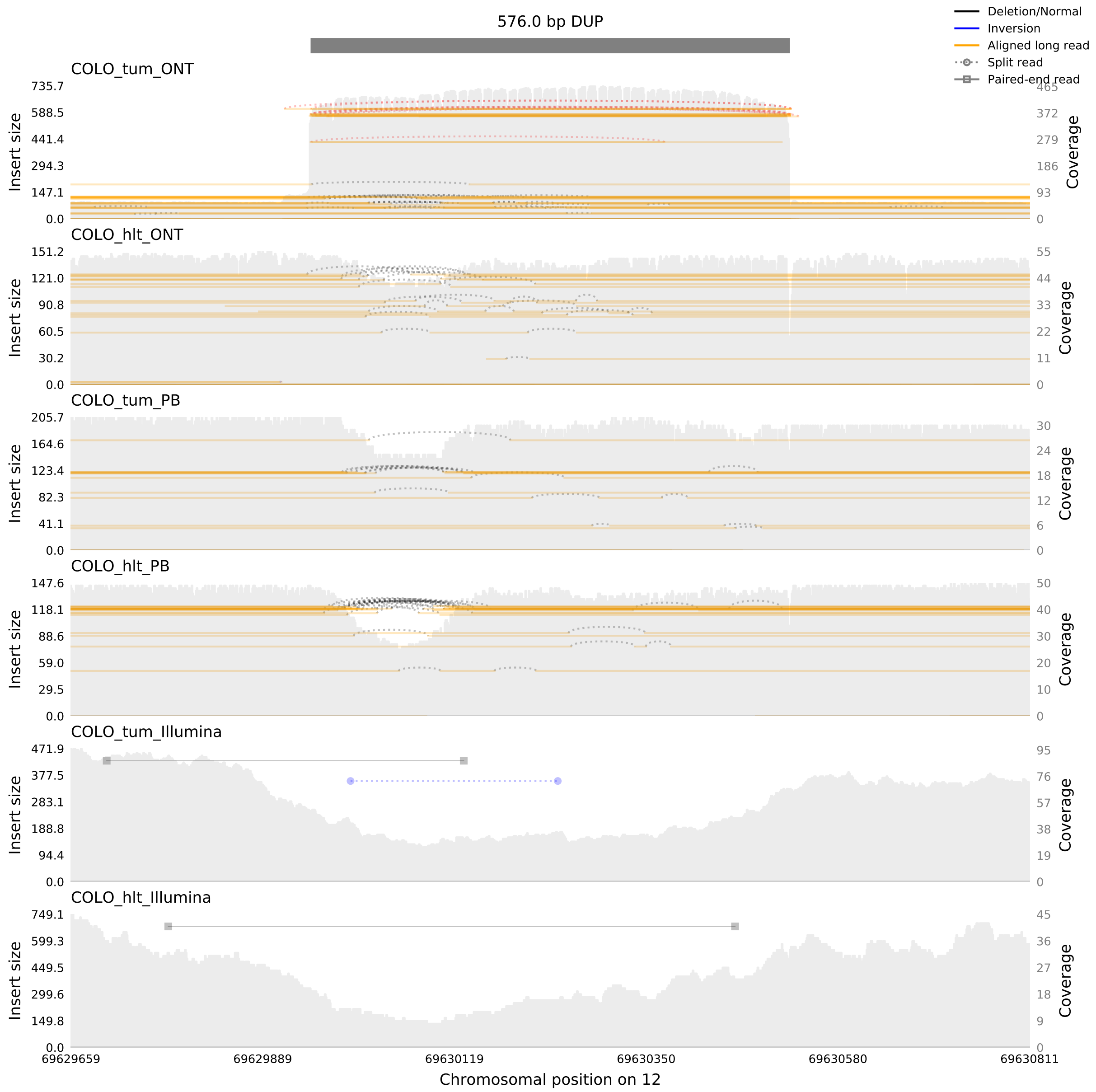

Supplementary Figure S32: Samplot of the 576 bb duplication detected by GASOLINE on COLO829 sample at 12:69629947-696305236. Reads are indicated by horizontal orange lines. Split alignments are indicated by color-coded dotted lines (black for deletions, red for duplications, blue for inversions) that allow to clearly and quickly distinguish between normal reads and reads supporting different SV types. The coverage for the region is shown with the gray-filled background, which is split into map quality above or below a user-defined threshold (in dark or light gray respectively). Split alignment signatures confirms the presence of the duplication in COLO829T and the absence in COLO829BL for ONT data. The variant is not present in both PacBio and Illumina data. The variant is considered false positive.

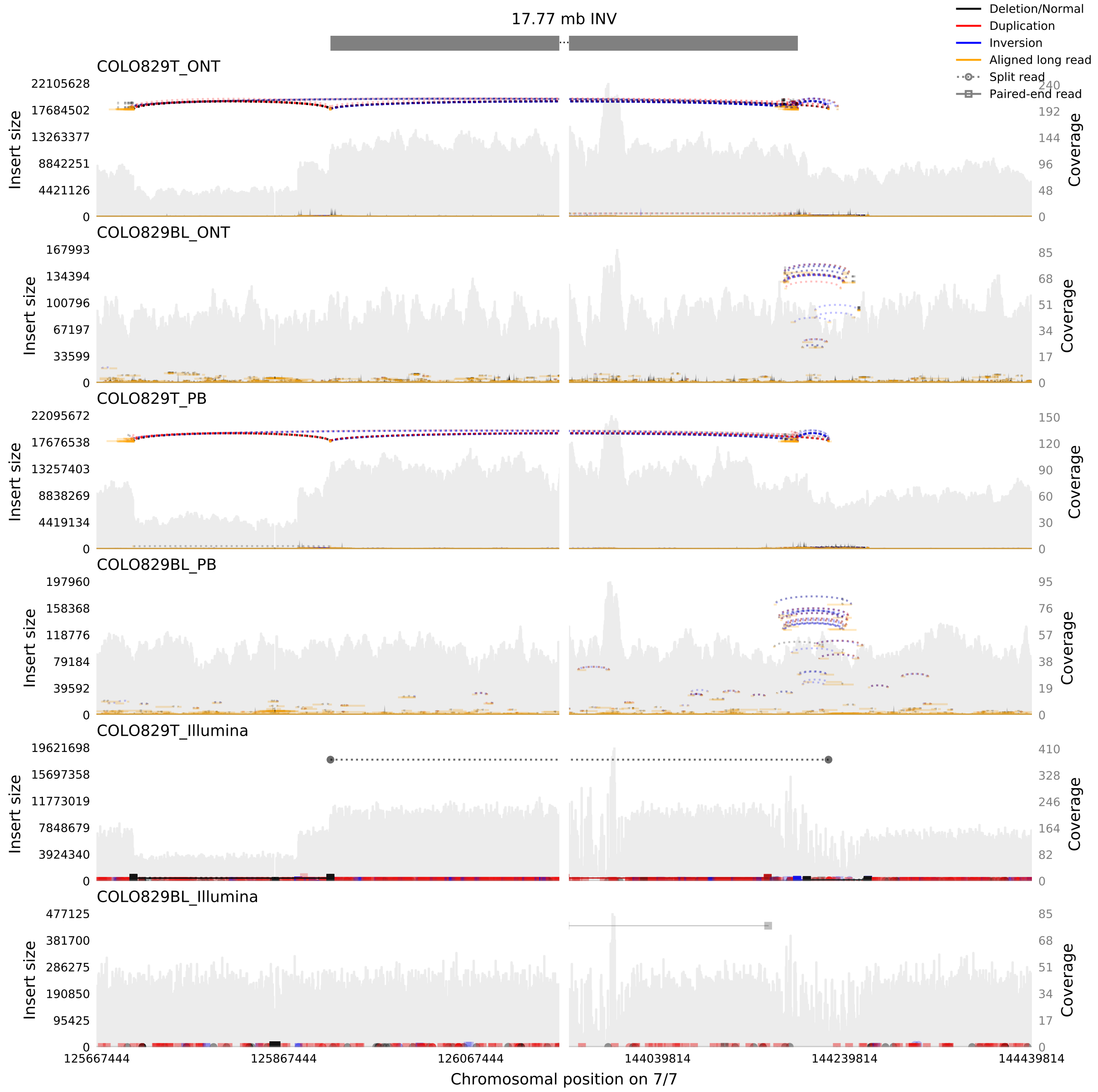

Supplementary Figure S33: Samplot of the 17.7 Mb inversion detected by GASOLINE on COLO829 sample at 7:126167444-143939814. Reads are indicated by horizontal orange lines. Split alignments are indicated by color-coded dotted lines (black for deletions, red for duplications, blue for inversions) that allow to clearly and quickly distinguish between normal reads and reads supporting different SV types. The coverage for the region is shown with the gray-filled background, which is split into map quality above or below a user-defined threshold (in dark or light gray respectively). Split alignment signatures confirms the presence of the duplication in COLO829T and the absence in COLO829BL for ONT, PacBio and Illumina data. The variant is considered true positive.

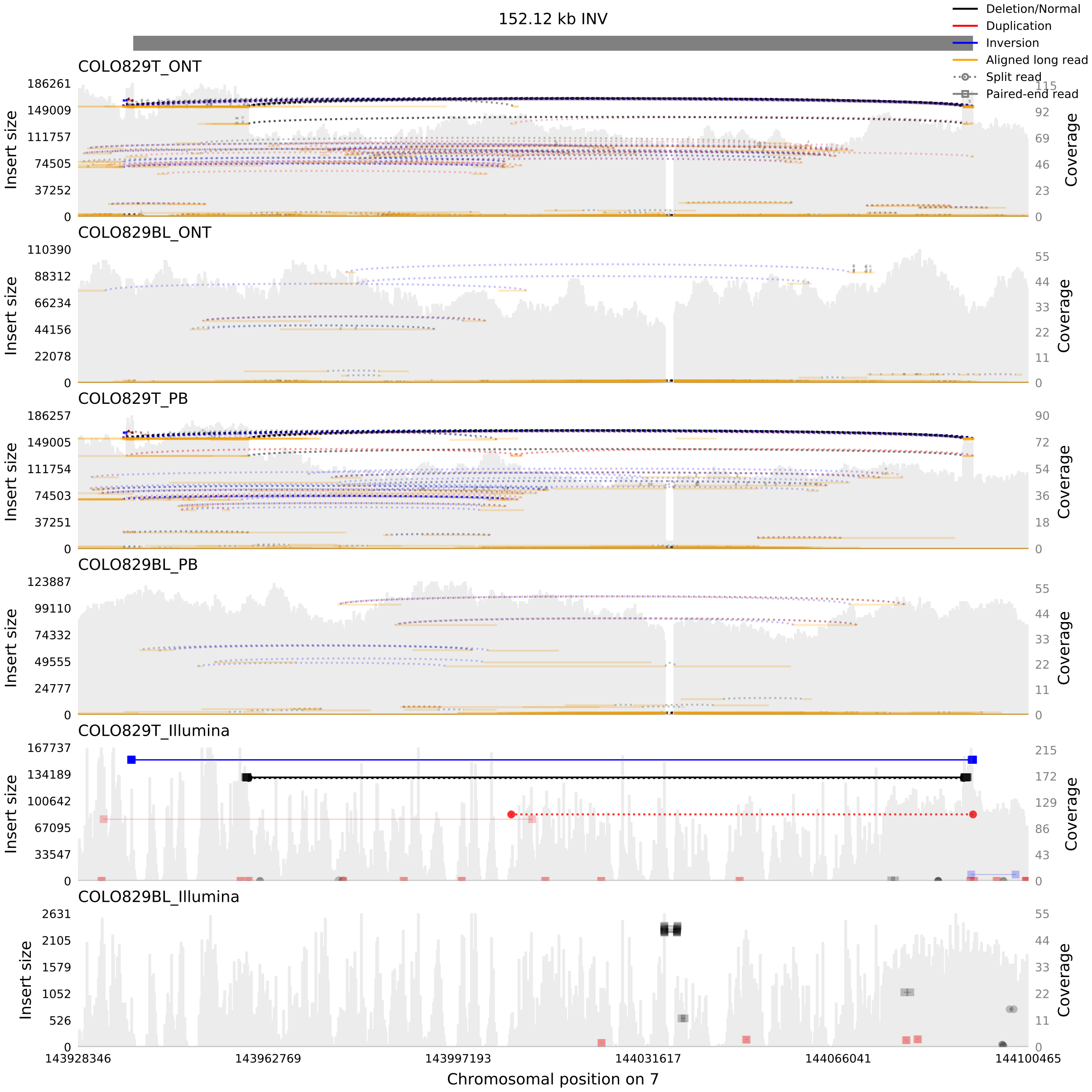

Supplementary Figure S34: Samplot of the 152 kb inversion detected by GASOLINE on COLO829 sample at 7:143936534-143937206. Reads are indicated by horizontal orange lines. Split alignments are indicated by color-coded dotted lines (black for deletions, red for duplications, blue for inversions) that allow to clearly and quickly distinguish between normal reads and reads supporting different SV types. The coverage for the region is shown with the gray-filled background, which is split into map quality above or below a user-defined threshold (in dark or light gray respectively). Split alignment signatures confirms the presence of the duplication in COLO829T and the absence in COLO829BL for ONT, PacBio and Illumina data. The variant is considered true positive.

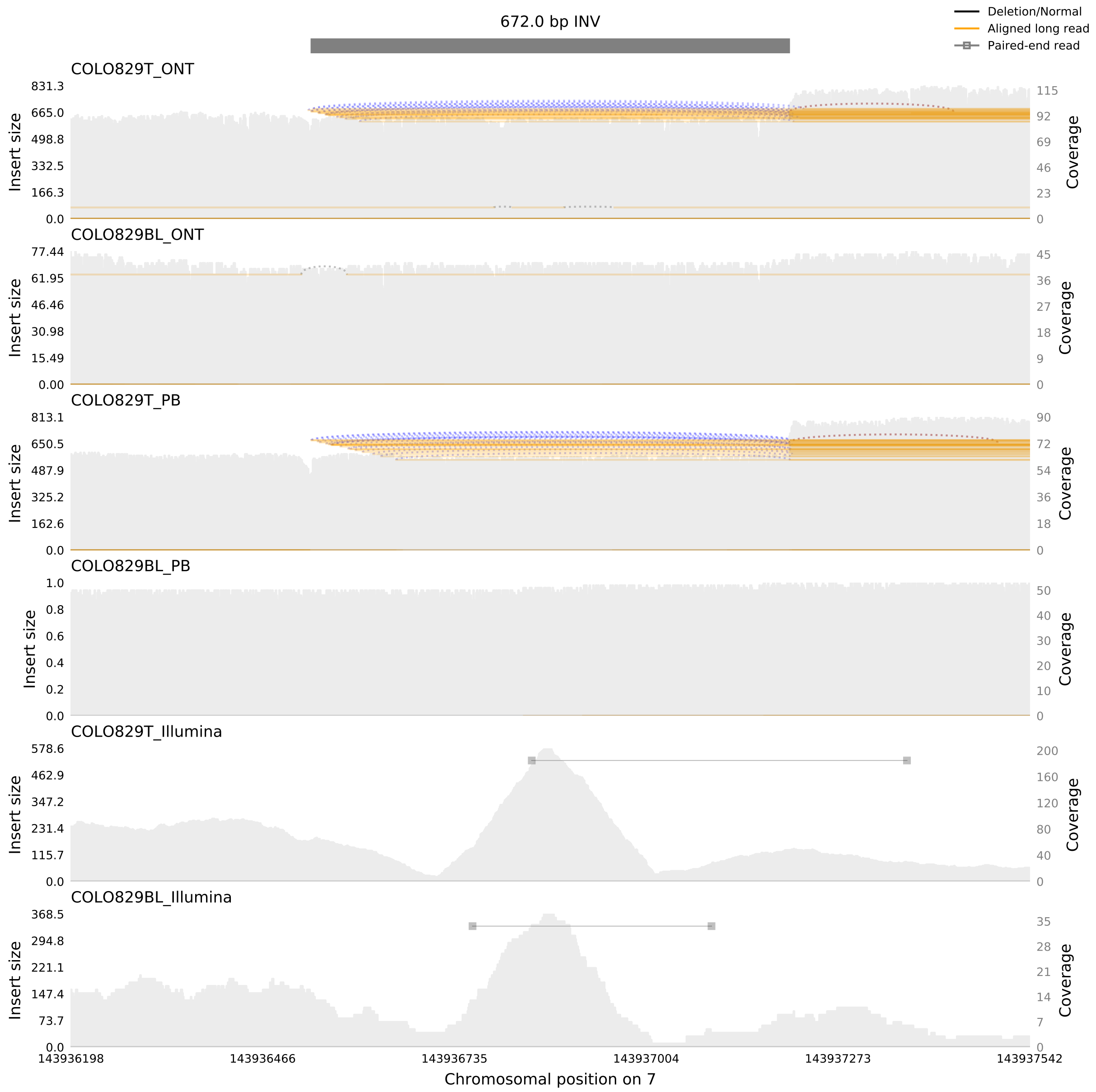

Supplementary Figure S35: Samplot of the 672 bp inversion detected by GASOLINE on COLO829 sample at 7:143936534-143937206. Reads are indicated by horizontal orange lines. Split alignments are indicated by color-coded dotted lines (black for deletions, red for duplications, blue for inversions) that allow to clearly and quickly distinguish between normal reads and reads supporting different SV types. The coverage for the region is shown with the gray-filled background, which is split into map quality above or below a user-defined threshold (in dark or light gray respectively). Split alignment signatures confirms the presence of the duplication in COLO829T and the absence in COLO829BL for ONT and PacBio data. The variant is considered true positive.
